# “Bringing in” and “Going abroad”: A bibliometric evaluation of the internationalization of archaeology in Mainland China

**DOI:** 10.1057/s41599-023-01800-0

**Published:** 2023-06-03

**Authors:** Xuan Wei, Wentai Lou, Ting Li, Ruxi Yang, Yinghua Li

**Affiliations:** 1 School of History, Wuhan University, Wuhan, 430072 China; 2 Archaeological Institute for Yangtze Civilization, Wuhan University, Wuhan, 430072 China; 3 Intellectual Computing Laboratory for Cultural Heritage, Wuhan University, Wuhan, 430072 China; 4 CNRS UMR 7041 ArScAn–AnTET, Université Paris X, Maison de l’Archéologie et de l’Ethnologie, 21 allée de l’Université, Nanterre, 92023 France

**Keywords:** Complex networks, History

## Abstract

Chinese scholars’ performance in international academic community and research on foreign archaeology has brought hot discussion about the internationalization of Chinese archaeology. Based on the databases of the China National Knowledge Infrastructure (CNKI) and the Web of Science core collection (WoS), this paper collected archaeology-related papers published by Chinese scholars in Chinese and world core journals (CCJs and WCJs for short), then filtered translated and original articles about foreign archaeology in CCJs, as well as all original archaeological articles in WCJs. Using Excel, CiteSpace and VOSviewer visualization software, we analyzed these data to give a bird’s-eye view of how archaeology research in Mainland China has become internationalized. Chinese archaeology has seen active-interrupt-active phases characterized by learning from foreign academics in the last century. Over the past two decades, the number of articles published in WCJs by scholars from Mainland China has increased significantly, and most research topics are at the forefront of international scholarship. Collaboration networks largely expanded, with the number of Mainland China–led articles increasing significantly. Archaeological papers written by researchers from Mainland China have appeared in a more extensive range of journals, including those with high impact factors. However, articles related to joint Sino-foreign archaeological projects were mostly published in CCJs. The archaeology-related articles published by Chinese scholars in WCJs occupied only a small proportion of all archaeological articles in WCJs. Compared to articles in CCJs, the number of those published by Chinese scholars in WCJs is a drop in the ocean. Therefore the internationalization is not yet a dominant trend and with the introduction of the new inward-looking policy we need more time to observe where the trends of internationalization and localization in Chinese archaeology are heading.

## Introduction

The association of Chinese archaeology with the international academic community dates back to the establishment of the discipline in the early 1920s. Before the Reform and Opening-up, such connections were sporadic, individual-based, and small scale, typically represented by the excavations in Yangshao by Swedish geologist J.G. Andersson in 1921; the excavations at the sites of Xiyin, Yinxu, and Zhoukoudian conducted by Ji Li, Siyong Liang, and Wenzhong Pei, respectively; as well as the collaborative excavations of China and North Korea in northeastern China (Wang 2017). Importantly, the internationalization of Chinese archaeology accelerated after the Reform and Opening-up in 1978. Several national policies were introduced, including the Measures for the Administration of Foreign-related Archaeological Work in the People’s Republic of China (NCHA 1991), the Going Out plan for the humanities and social sciences in universities (MOE 2011), the Belt and Road Initiative (BRI for short, NDRC et al. 2015), and President Jinping Xi’s speech at the symposium on philosophical and social science work (Xi, 2016), as well as his call for advancing study of Chinese civilization (Xi 2022).

The Center for Foreign Archaeological Research at the Institute of Archaeology, Chinese Academy of Social Sciences (IA CASS, see Supplementary Table S1 online for all abbreviations used in this article) was established in 2017 to help internationalize Chinese archaeology (Storozum and Li 2020), and manifestations of internationalization thus became more diverse. At the domestic level, this included cooperation with foreign institutions from the United States and Japan to excavate essential sites, hold international conferences, and translate foreign archaeological research results for publication in domestic journals. At the international level, national or local archaeological institutions or universities conducted archaeological fieldwork in other countries; a large number of scholars studying abroad for degrees participated in international academic conferences, holding positions in international educational organizations, and publishing research results in international journals, among other activities (Wang 2017). The internationalization of Chinese archaeology has become a topic of discussion among scholars (Storozum and Li 2020; Wang 2017; Yuan 2017), but only from a qualitative perspective, and no attempt has been made to quantitatively analyze this discourse.

In the past few decades, the development of quantitative methods has given rise to a new discipline, information science, which is composed of three overlapping fields: bibliometrics, scientometrics, and info-metrics (Hood and Wilson 2001; Leydesdorff 2001; Osareh 1996a, b). As a widely accepted technique, the bibliometric method—particularly mathematical and statistical approaches—has been applied to all types of scholarly communication in the natural and social sciences (Pritchard 1969). It has also been proven effective in describing and evaluating the history and performance of individual disciplines, institutions, and even countries in terms of scholarly production and knowledge diffusion (Potter 1988). At present, the increasing application and proliferation of big data in e-resource databases—including the China National Knowledge Infrastructure (CNKI), published by Tsinghua University, and the Web of Science core collection (WoS), published by the American Institute for Scientific Information (ISI) —have further facilitated the use of quantitative assessment indicators and tools developed based on bibliometrics to evaluate the performance of academic disciplines, institutions, and countries in a more visual way.

In the field of archaeology, bibliometric methods have also been used to review the discipline or a subfield’s development (Lin 2016; Mays 2010; Palomar et al. 2009; Pena-Poza et al. 2011) or to discuss some special topics (Bardolph and Vanderwarker 2016; Beaudry and White 1994; Beck et al. 2021; Heath-Stout (2020); Hutson 2002, 2006; Sterud 1978). Particularly, this method has recently been applied to make a macroscopic review of the general characteristics and trends of Chinese archaeology on the basis of the papers indexed in CNKI and WoS datasets during the past century (Wei et al. 2023). By adding new data published in 2021 into the dataset and using new software, i.e. Citespace and VOSviewer, in addition to Microsoft Excel, this paper focuses on how archaeology research in China has become internationalized and how is the current level of internationalization of Chinese archaeology, which means a more focused and in-depth bibliometric study. Actually, research on the internationalization of academic disciplines from a bibliometric perspective has been well established for years (Boncourt 2018; Matveyev and Savelieva 2017; Sivertsen 2016; Zhang et al. 2020; Zhang et al. 2018). Theoretically, internationalization consists of three interrelated levels: national, institutional, and individual (Zhang et al. 2020). In this framework, substantial papers published by individual scholars in academic journals—containing rich and easily quantifiable information—have become the preferred data source for analyzing disciplinary internationalization; the indicators for evaluation usually include countries’ distributions of references, different methods for international collaboration, research content of global issues, journals, and publication impact (Zhang et al. 2020). So as a first attempt, the current study focuses on research content, international issues, international collaboration, and journals of publication, based on translated articles and original articles on foreign archaeological research in Chinese core journals (CCJs) and all articles published by Chinese archaeologists in World core journals (WCJs). We collected all archaeology-related articles in WCJs published by scholars of mainland China, Hong Kong, Macao, and Taiwan, but in the analysis, if not specifically stated, we focus on Mainland China while presenting and analyzing the data from Hong Kong, Macao, and Taiwan separately.

The following research questions are of significant concern in this study:

1. How well were Chinese archaeologists informed about the international archaeological community in the last seven decades?
2. How well did Chinese archaeology internationalize in terms of general performance and degree of coupling between Chinese archaeological research and international academic frontiers?
3. What is the extent of international collaboration in Chinese archaeological research?
4. What are the characteristics of the journals in which Chinese scholars have published archaeological papers?

Following these questions, the internationalization of Chinese archaeology is briefly reviewed, the degree thereof is assessed, and an outlook on trends is provided.

## Data and method

### Data source

We collected all archaeology-related papers from the two largest databases of scientific literature accessed via the library of Wuhan University: CNKI in China and the global WoS core collection.

In CCJs, we collected papers based on the CNKI e-resources database. Several citation indexes for sciences, social sciences and humanities were established and largely accepted in China; they are Chinese Sciences Citation Database (CSCD for short), Chinese Social Sciences Citation Index (CSSCI for short), and Chinese Core Periodicals of PKU (CCP-PKU for short). So, all the journals indexed by these datasets (according to CSCD 2020, CSSCI 2019–2020 and CCP-PKU 2020) were firstly included in the analysis. In view of the interdisciplinary nature of archaeology, we not only collected all the journals belonging to the “Archaeology” catalog but also filtered the journals belonging to the “History” and “Geology” catalog. In sum, we collected 27 Chinese Core Journals in total (see Table 1 for details). It should be noted that some journals such as *Archaeological Collectanea* (Kaoguxue Jikan), *A Collection of Studies on Archaeology* (Kaoguxue Yanjiu), *East Asia Archaeology* (Dongfang Kaogu), *Beijing Cultural Relics and Museums* (Beijing Wenbo), etc. are also related to archaeology, but they are not included in the three indexes mentioned above, so they are not included in our data source.

**Table 1:** List of Chinese core journals.

Chinese core journals (CCJs)	Number of articles	Time of the First Issue	JIF quartiles
Archaeology 考古	8045	1955	Q1
Cultural Relics 文物	4437	1950	Q1
Cultural Relics of Central China 中原文物	3169	1977	Q1
Archaeology and Cultural Relics 考古与文物	3036	1980	Q2
Cultural Relics in Southern China 南方文物	2906	1962	Q2
Jianghan Archaeology 江汉考古	2626	1985	Q1
Southeast Culture 东南文化	2314	1980	Q1
Huaxia Archaeology 华夏考古	1868	1987	Q2
Sichuan Cultural Relics 四川文物	1844	1984	Q3
Relics and Museolgy 文博	1680	1984	Q3
Northern Cultural Relics 北方文物	1460	1981	Q3
Acta Anthropologica Sinica 人类学学报	1230	1982	Q3
Acta Archaeologica Sinica 考古学报	1125	1936	Q1
Journal of National Museum of China 中国国家博物馆馆刊	1052	1979	Q2
Agricultural Archaeology 农业考古	978	1981	Q3
Research of China’s Frontier Archaeology 边疆考古研究	773	2002	\
Palace Museum Journal 故宫博物院院刊	412	1958	Q2
Sciences of Conservation and Archaeology 文物保护与考古科学	370	1989	Q2
Dunhuang Research 敦煌研究	325	1981	Q2
Quaternary Sciences 第四纪研究	318	1958	Q2
Southern Ethnology and Archaeology 南方民族考古	287	1987	\
Journal of Dunhuang Studies 敦煌学辑刊	154	1983	Q1
Vertebrata Palasiatica 古脊椎动物学报	149	1957	Q4
Historical Research 历史研究	111	1954	Q1
Chinese Science Bulletin 科学通报	94	1950	Q1
Scientia Sinica(Terrae) 中国科学·地球科学	57	1996	Q1
Social Sciences in China 中国社会科学	45	1980	Q1
**Total**	**40,865**		

The JIF quartiles were derived from the *Annual Report for Chinese Academic Journal Impact Factors (Social Science) (2022)* published by CNKI (CSBRC, TUL 2022).

The papers in more than 500 WCJs were collected from the Web of Science (WoS) Core Collection, including the Science Citation Index (SCI), the Social Sciences Citation Index (SSCI), and the Arts & Humanities Citation Index (A&HCI) (see Table 2 for details).We used a series of keywords for the search, including “archaeolog*,” “Paleoanthropolog*,” “Prehistor*,” “Paleolithic*,” “Neolithic*,” and selecting “Peoples R China,” “China,” “Hong Kong,” “Macao” or “Taiwan” for “Affiliation” and “Article” for “Document Type”. The publication is included in our data set for analysis if the institutional address of at least one of the authors is linked to China, Hong Kong, Macao, or Taiwan. Notably, the data analyzed in this paper only included papers directly related to archaeology *sensu stricto*, not museums, cultural heritage, cultural relics protection and management, or ancient architecture. The deadline for data collection was December 31, 2021 (articles *in press* were also included).

**Table 2:** List of World core journals publishing archaeology-related articles of Chinese scholars.

World core journals (WCJs)	Number of articles	Time of the First Issue	JIF quartiles
Quaternary International	274	1989	Q2
Journal of Archaeological Science	152	1974	Q1
Archaeometry	100	1958	Q2
Chinese Archaeology	96	2001	ESCI
Plos One	91	2006	Q1
Chinese Science Bulletin	91	1956	Q2
Holocene	88	1991	Q2
Antiquity	86	1927	Q1
Scientific Reports	69	2011	Q1
Journal of Archaeological Science-Reports	65	2014	A&HCI
Quaternary Science Reviews	60	1982	Q1
Proceedings of the National Academy of Sciences of the United States of America	58	1915	Q1
Science China-Earth Sciences	57	1996	Q2
Archaeological and Anthropological Sciences	53	2009	Q1
Journal of Human Evolution	52	1972	Q1
Archaeological Research in Asia	50	2014	A&HCI
Anthropologie	49	1890	Q4
American Journal of Physical Anthropology	47	1918	Q1
Nature	46	1869	Q1
Quaternary Geochronology	42	2006	Q2
Quaternary Research	40	1970	Q2
Palaeogeography Palaeoclimatology Palaeoecology	39	1965	Q1
International Journal of Osteoarchaeology	38	1991	Q1
Spectroscopy and Spectral Analysis	35	1981	Q4
Bulletin of the Institute of History and Philology Academia Sinica	29	1928	A&HCI
Vegetation History and Archaeobotany	25	1992	Q1
Science	25	1880	Q1
Journal of Cultural Heritage	25	2000	Q1
Molecular Biology and Evolution	24	1983	Q1
Journal of Geographical Sciences	23	1990	Q2
Radiocarbon	22	1959	Q2
Journal of Anthropological Archaeology	21	1982	Q1
Nuclear Instruments & Methods in Physics Research Section B-Beam Interactions with Materials and Atoms	20	1984	Q3
**Others**	**1190**		
**Total**	**3182**		

This table only shows the journals publishing more than 20 archaeology-related articles. The JIF quartiles are derived from the website of Journal Citation Report: https://jcr.clarivate.com, the same as below.

### Data processing and methods

**Fig. 1:**
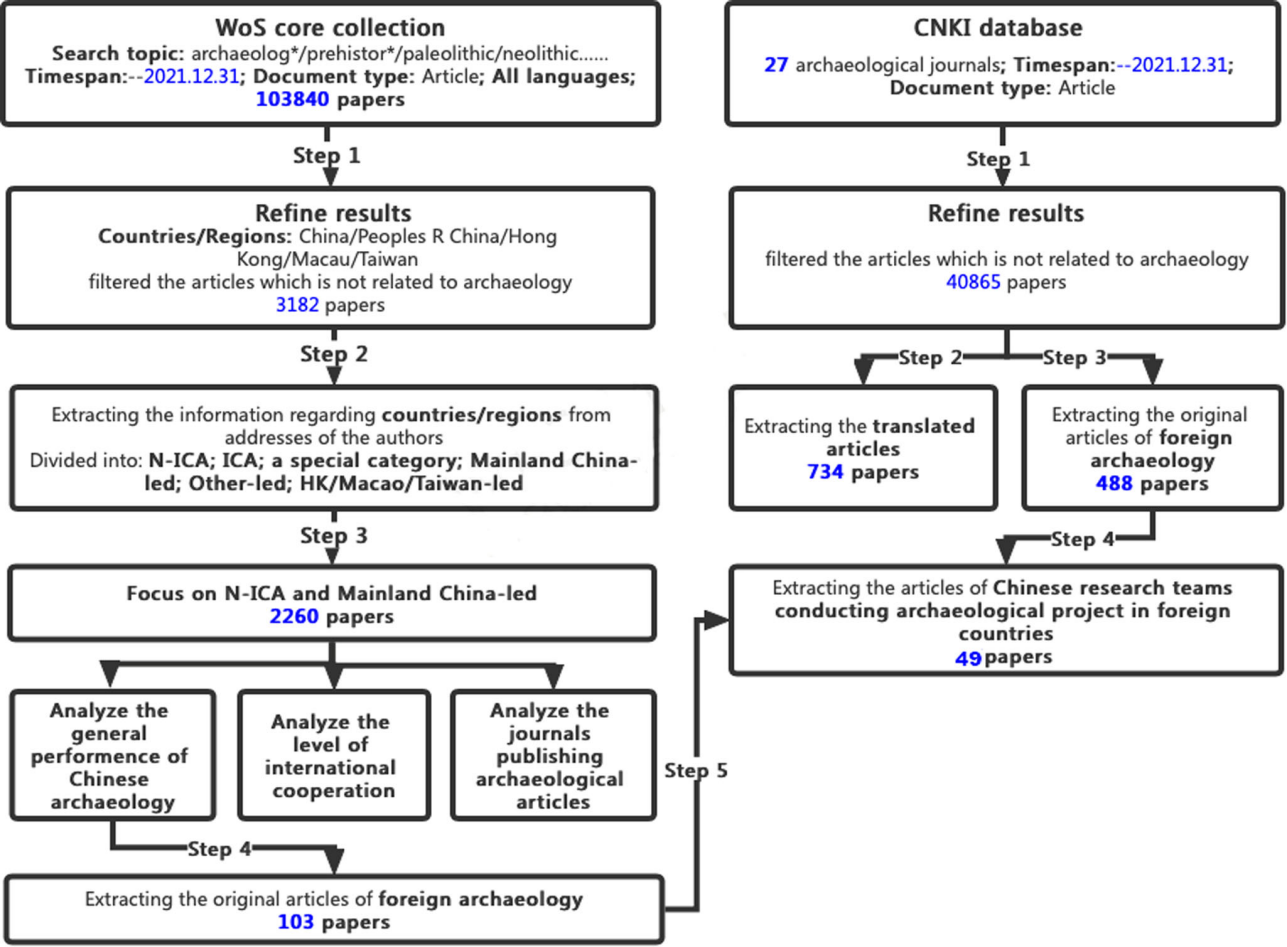
Data source and data processing. On the left, data are collected from the WoS database and then refined and classified through five steps. On the right, data are collected from the CNKI database and then refined and classified through four steps.

According to the research content and aims of this work, we organized the data source and processing procedures, as indicated in Fig. 1. The raw data were examined and calculated by using various software, including Excel (Microsoft 365), VOSviewer (version 1.6), and CiteSpace (version 6.1 R6), each having its own strengths. The latter two are visualization mapping software and are frequently used in current bibliometric analysis. On a technical level, VOSviewer pays special attention to the co-occurrence of keywords, authors, or institutions in a graphical representation of bibliometric maps, which are especially useful for displaying data in an easy-to-interpret way (Eck and Waltman 2010); CiteSpace is slightly better to generate interactive visualizations of structural and temporal patterns and trends of a scientific field by mapping timeline and time-zone views (Chen 2006).

Note, we only collected the papers in which the first or corresponding author’s affiliation is in Mainland China because the order in which the authors’ names appear generally reflects the contribution that each author made to the article (Egghe et al. 2003), with the first and corresponding author typically playing a leading role in the research and writing process (Lariviere et al. 2016).

#### Screening of translated articles published in CCJs

The translated articles were filtered from the CNKI and organized by the number of the annual publication. We then divided these translations into three categories based on the research content: foreign archaeological research, foreign archaeological theory and method, and Chinese archaeological study conducted by foreign researchers. The countries of origin of the translated articles and the journals publishing them were particularly considered.

#### Screening of original articles on foreign archaeology in CCJs and WCJs published by Mainland Chinese scholars

We screened the original articles on foreign archaeology (AFAs for short, i.e., the articles about research on foreign sites or remains, not including translated articles and abstracts) published by Chinese scholars in CCJs and WCJs. We then tabulated the data by the number of annual publications and the countries concerned. At the same time, we highlighted the articles directly related to Sino-foreign archaeological projects that Chinese teams conducted in foreign countries.

We also analyzed all original articles on archaeology published by Mainland Chinese scholars in WCJs. The high-frequency occurring keywords of these articles were examined with the software CiteSpace. We chose “keyword” in the “Node types”, “from 1980 to 2021” in “Time Slicing”, set “#Years Per Slice” to “5” and others to “default value”. To better understand the relationship between hot topics of research among Chinese archaeologists and the international archaeological community, we compared the high-frequency occurring keywords between domestic and international scholar publications in WCJs. All archaeology-related publications searched from WoS (not refined by countries, 103,840 papers in total) were loaded into CiteSpace for the keywords analysis, with the parameters set as above; we then compared these keywords with those in articles published by Mainland Chinese researchers.

Then the international influence of Chinese authors was analyzed by using the software CiteSpace, from the two sections of highly published and highly cited authors. To analyze the highly published authors, we chose “Author” in the “Node Types”, and set other parameters as above, while in the analysis of highly cited authors, “Cited Author” is chosen in the “Node Types”. The Time-Zone view of CiteSpace shows the result of highly cited authors in the different stages.

#### Types of collaboration reflected in WCJ articles

**Fig. 2:**
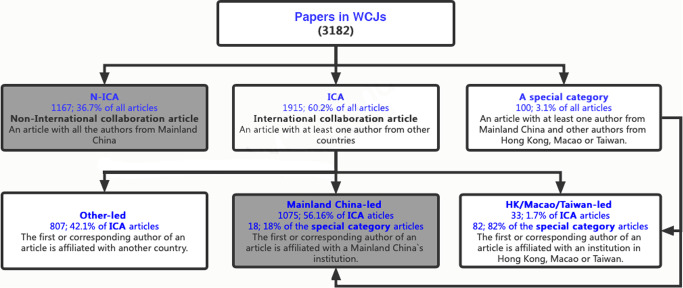
Types of collaboration between Chinese and foreign institutions (the shaded parts are considered in this article; revised according to Zhang et al. 2020). The WCJ papers can be classified into three groups according to the institutions of authors: N-ICA, ICA, and A special category (Hong Kong, Macao, Taiwan). The ICA group can be further divided into three subgroups based on the location of the first or corresponding author’s institution. Similarly, a special category group can be subdivided into Mainland China-led and HK/Macao/Taiwan-led.

To conduct an in-depth analysis of international collaboration, we identified and defined two types of articles: non-international collaboration articles (N-ICAs) in which all researcher affiliations are in Mainland China, and international collaboration articles (ICAs) in which researcher affiliations are in different countries/regions and at least one researcher is from Mainland China. Because this paper focuses on the internationalization of archaeology in Mainland China, we did not include articles in which all authors are from Hong Kong, Macao, or Taiwan; we also analyzed articles with at least one author from Mainland China and other authors from Hong Kong, Macao, or Taiwan separately. To understand the role of Chinese researchers in international collaboration, we further divided the articles into three types according to the affiliation of the first/corresponding author. Mainland China-led ICA refers to papers in which the first or corresponding author is affiliated with Mainland China. Other-led ICA refers to articles in which the first or corresponding author is from another country. Hong Kong/Macao/Taiwan-led ICA includes the articles in which the first or corresponding author is from institutions in Hong Kong, Macao, or Taiwan. Figure 2 illustrates how we processed the data in WCJs and classified different types of collaboration.

We then used the dual-map overlay visualization provided by VOSviewer to conduct institutional co-authorship analysis on N-ICAs and Mainland China-led ICAs to display the institutional collaboration networks suggested by co-authored articles. The “type of analysis” and “unit of analysis” were respectively set to “co-authorship” and “organization,” and the “counting method” was set to “all counting” in the software parameters. The generated dual-map overlay visualization overlaid the institution’s average publication year (i.e., the average between the timing and number of articles over years). Note that the number of institutions and their publications obtained from this analysis may be much higher than the actual number of articles because the co-authors of an article are often affiliated with multiple organizations.

#### WCJs publishing archaeological articles

To obtain a bird’s-eye view of international journals publishing archaeological articles and the performance of Mainland China in these journals, we examined journal distribution across disciplines, countries of publication, journal quartiles, and collaboration types by sorting the journal articles in which Mainland Chinese researchers appeared as the first or corresponding authors. The Journal Citation Reports (JCR) website provides all journal information used in this work, and the journal disciplines were sorted by WoS Category (WC). Because one journal is often affiliated with more than one discipline, the number of journals counted may be greater than the actual number of journals, as well as their disciplines. We also ranked the journal impact factor (JIF) quartiles of these international journals based on the JCR of 2021. In cases when a journal belonged to more than one WC, we used the best quartile result of that journal in that year. It should be noted that, in JCR, the JIF quartiles are only for SCI and SSCI, but not for ESCI and A&HCI, so we directly marked ESCI or A&HCI for journals included in ESCI and A&HCI.

## Results

We collected a total of 44,047 papers about archaeology, including 40,865 papers from CCJs and 3182 papers from WCJs. After filtering, our sample consisted of 734 translated articles, 591 original articles of foreign archaeological research, and 2260 archaeology-related articles published by Chinese scholars in WCJs (i.e., the first or corresponding author is affiliated with a Mainland Chinese institution).

### Knowledge level of the international academic community

Translating articles from foreign countries is a fundamental means for understanding advances in and the status of foreign archaeology. It also reflects the willingness of domestic scholars to integrate into the international academic community. Therefore, we analyzed all translated articles to investigate the diachronic change, the categories of content, distribution of countries of origin of the translated articles to journals publishing them.

#### Contribution of translated articles published by Chinese archaeologists

**Fig. 3:**
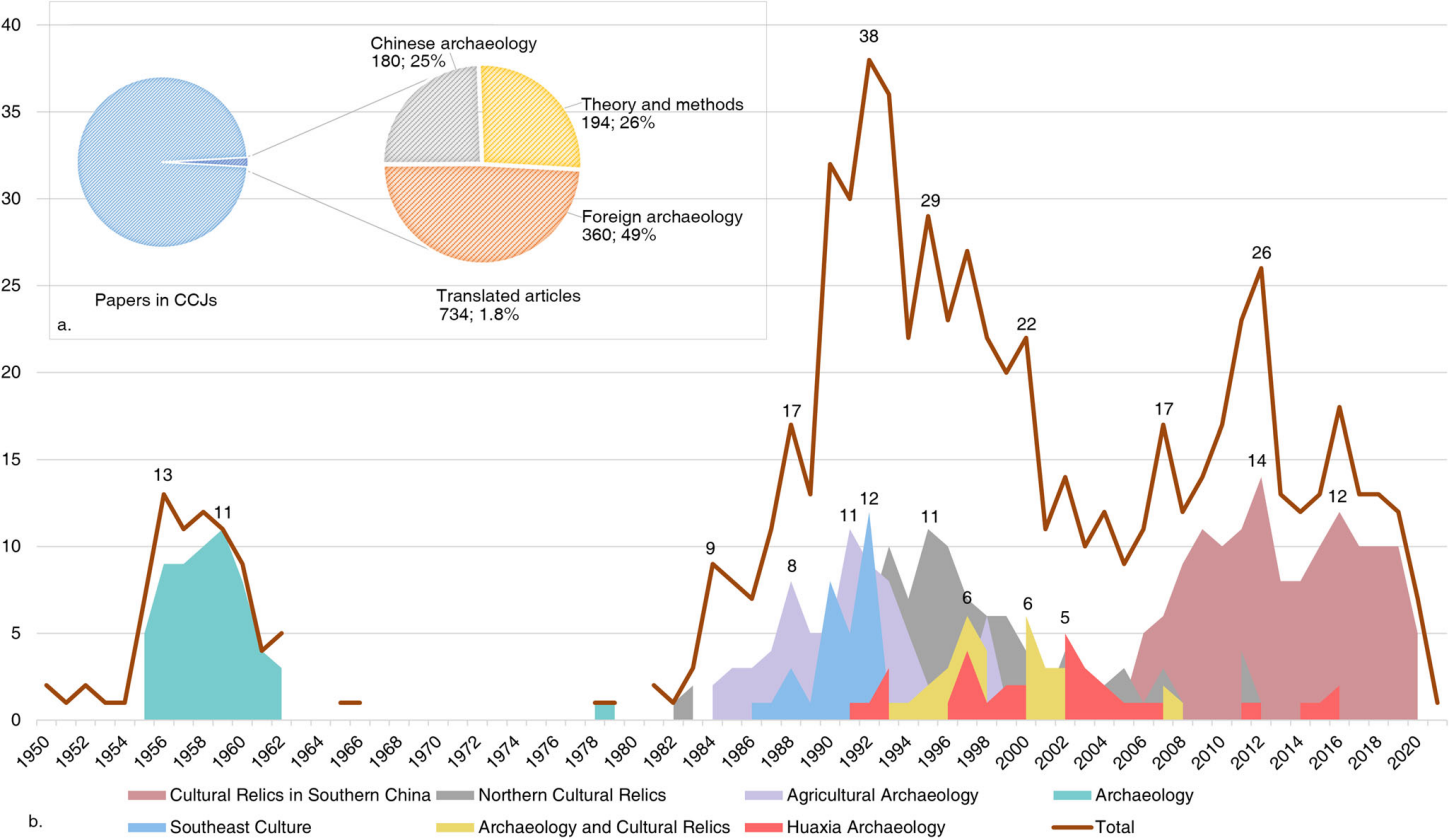
Translated articles. **a** Proportion of translated articles in total articles in CCJs and respective percentages of different types of translated articles. **b** Annual change curve of all translated articles and of translated articles published in each journal.

A total of 734 translated articles were collected, accounting for only 1.8% of all Chinese archaeological papers in CCJs (Fig. 3a). The annual translated article volume curve (Fig. 3b) displayed the “twists and turns” of the curve starting from 1950; the late 1950s to the early 1960s was the first peak, when most of the translated articles were published in *Archaeology*, one of the top journals in Chinese archaeology. But this progress was almost entirely interrupted in the 1960s and 1970s, and it did not develop again until the 1980s. The second peak for publishing translated articles occurred during the 1980s to 2000. Some journals stood out as the main fronts for publishing them, including *Northern Culture Relics*, *Agricultural Archaeology*, *Southeast Culture*, and *Archaeology and Cultural Relics*. In the early twenty-first century, the number fell back slightly. While in the last decade the third peak of translated articles re-occurred, most of which were published in the *Cultural Relics in Southern China* during this period, especially after 2005.

In terms of content, translated articles were divided into three categories, with the proportion of each category showing some degree of variation (Fig. 3a). Original articles about foreign archaeology received the highest attention (49%), followed by translated articles about archaeological theory and methods (26%). It is also noteworthy that a significant proportion of the translated articles are on Chinese archaeological research done by foreign scholars (25%).

#### Country-wise distribution of translated articles

The country-wise distribution of the original author of translated articles reflects the extent to which different foreign academic schools have influenced the Chinese archaeological community. We counted the number of original authors of translated articles, ranked the top six by country of origin of them, and analyzed the distribution of them to different journals (Table 3). The original authors of translated articles come from 40 different countries around the world, nearly a third coming from Japan, which is very close to China; most of these translated articles are on studies of ancient Chinese culture and remains. In second place was the United State. The extremely high interest in archaeology of the Soviet Union reflects the political context in the 1950s and 1960s. There are also a certain number of translated articles written by authors from other countries, such as the United Kingdom, Canada, Australia, Denmark, France, Russia, and other European countries, which indicates that Chinese archaeology has a wide range of sources of study and understanding within foreign scholarship.

**Table 3:** Distribution of countries of origin of the translated articles to the journals publishing them.

Journal	Japan	USA	Soviet Union	England	Russia	Canada	Others	Total
Cultural Relics in Southern China	22	77	0	21	1	19	25	165
Northern Cultural Relics	34	2	19	0	42	10	3	110
Agricultural Archaeology	30	29	1	2	0	4	11	77
Archaeology	6	4	41	1	0	0	24	76
Archaeology and Cultural Relics	20	2	0	9	1	1	6	39
Southeast Culture	15	13	0	2	0	4	5	39
Huaxia Archaeology	20	9	0	1	0	1	5	36
Others	62	52	15	16	4	0	2	2
**Total**	**209**	**188**	**76**	**52**	**48**	**39**	**70**	**734**

It only shows the countries of origin and journals having more than 30 translated articles.

In terms of journals publishing translated articles, *Cultural Relics in Southern China* was ranked first and the majority of original authors comes from the USA, as shown in Table 3. The second was *Northern Cultural Relics* and most of the translated articles were written by authors from Japan, the Soviet Union, and Russia——mainly the Northeast Asian countries. And *Arch*aeology published the largest number of translations of articles whose original authors were from the Soviet Union and other Socialist countries. While other journals, such as *Agricultural Ar*c*haeology*, *Southeast Culture*, and *Huaxia Archaeology* published large number of translations of articles from Japan and the USA.

In terms of the influence of these journals, as shown in Table 1, *Archaeology* and *Southeast Culture* are ranked Q1, highly influential journals in the field of archaeology (*Southeast Culture* is listed in “history” catalog) in China, each of them focusing on different regions. The *Cultural Relics in Southern China*, which published the most translated articles, as well as *Archaeology and Cultural Relics* and *Huaxia Archaeology* belong to Q2. While the *Northern Cultural Relics* and *Agricultural Archaeology*, which published the second and third most translated articles, respectively, belong to Q3. In sum, most of the journals that published translated articles and focused on foreign archaeological research are not listed as the most influential journals, but they are important for showing to Chinese researchers the diversity of archaeological research around the world.

### Involvement level of Chinese archaeologists in the international academic community

Original research conducted by Chinese archaeologists on foreign archaeological sites and materials (i.e., “going abroad”) is an important indicator of the extent to which Chinese archaeology is internationalized. In this part, we investigated the general performance of the internationalization of Chinese archaeology from four perspectives: the general character of the AFAs primarily related to joint Sino-foreign archaeological fieldwork; changes in archaeological articles published by Mainland Chinese scholars in WCJs over time; the development trend of research theme over time and the degree of coupling between Mainland Chinese archaeological research and the international academic community; and the influential Chinese archaeologists in different stages.

#### AFAs published by Chinese archaeologists

**Fig. 4:**
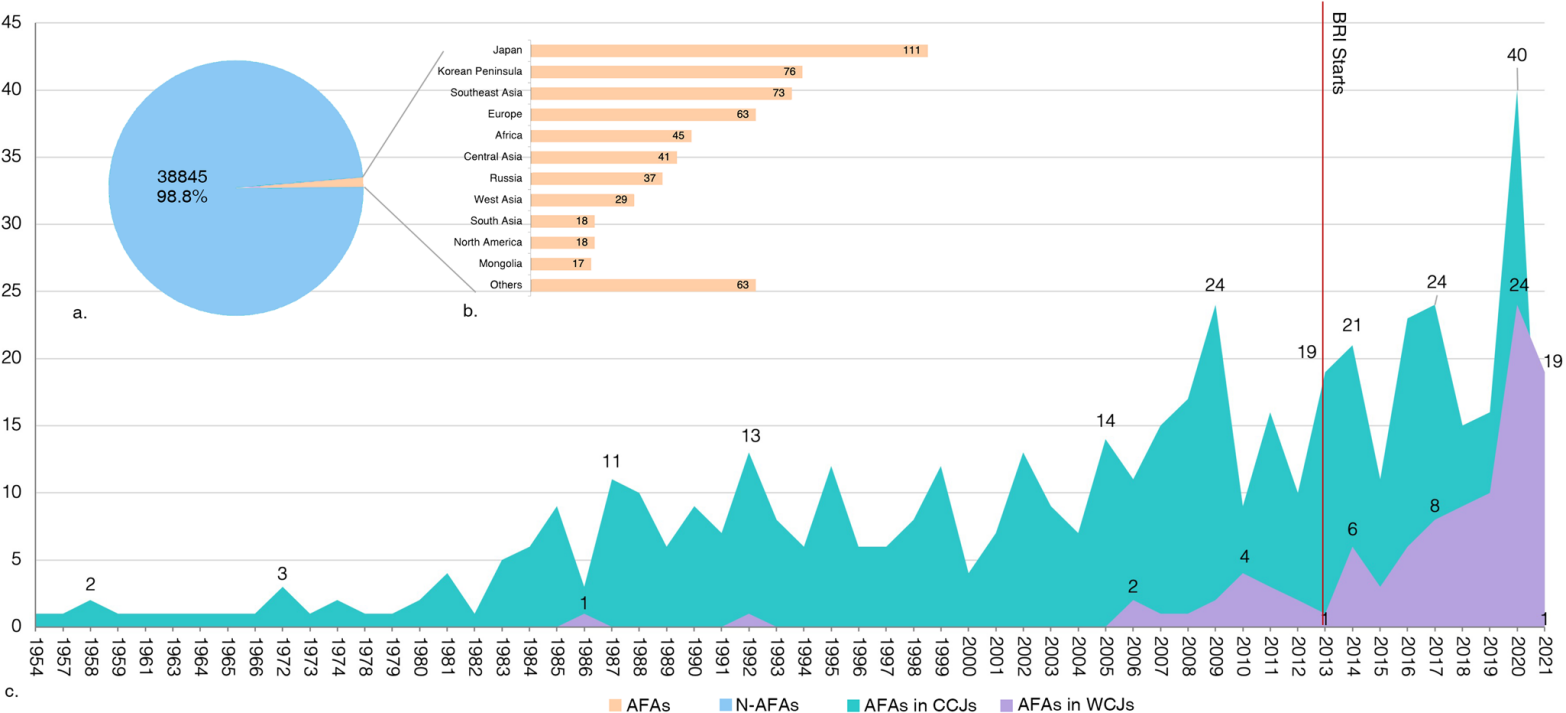
Articles on foreign archaeology (AFAs). **a** Proportion of AFAs and non-AFAs in WCJs. **b** Distribution of countries involved. **c** Change of AFAs over time.

A total of 591 papers of AFAs were screened, accounting for only 1.24% of all archaeological papers, in which 488 papers in CCJs, accounting for 1.2% of all CCJs papers, while 103 papers were published in WCJs, accounting for 4.6% of all WCJs papers (Fig. 4a). The vast majority of articles on foreign archaeology focused on countries adjacent to China——Japan, Korea, Russia, Southeast Asia, and Central Asia—with some attention to Europe and Africa, but little to West Asia, North America, and other regions (Fig. 4b).

The change in publication numbers over years (Fig. 4c) indicates that the AFAs first appeared in CCJs in 1954, but only in a very small number. After the 1980s, AFAs increased and began to appear in WCJs. Since the twenty-first century—especially since BRI was launched in 2013— AFAs have increased significantly, with the fastest growth in WCJs; the number of AFAs in WCJs is far more than CCJs in 2021 (19 in WCJs while 1 in CCJs).

We then screened 49 articles directly related to Chinese-led collaborative archaeological projects conducted in foreign countries, accounting for 8.3% of all AFAs. It may be noteworthy that among them 45 papers were published in Chinese and only 4 in other languages. The 49 articles and list of joint Sino-foreign archaeological projects can be found in Supplementary Table S2 online. In terms of the document type, there were 33 excavation reports and 16 original research articles. Because only 27 CCJs were counted and only SCI/SSCI/A&HCI-indexed journals were included in our work, a very small number of AFAs may have been omitted.

#### Periodic development of publications in WCJs

**Fig. 5:**
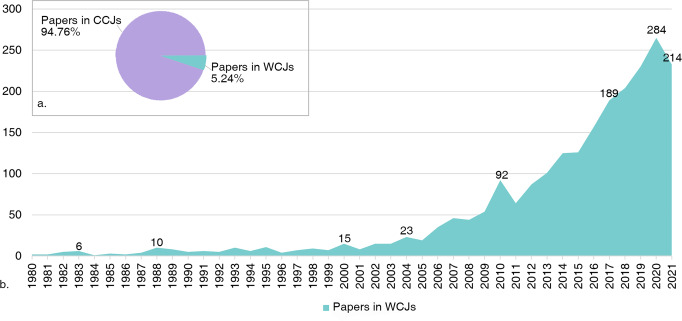
Articles on WCJs. **a** Percentage of articles published by Chinese scholars in WCJs among all articles published by Chinese scholars. **b** Annual change curve of archaeology-related articles published by Chinese scholars in WCJs.

A total of 2260 original archaeological articles with the first or corresponding authors from China were collected from WCJs, accounting for 5.24% of all archaeological articles published by Chinese scholars in both CCJs and WCJs (Fig. 5a). A research review about Chinese Neolithic archaeology written by Zhimin An in Chinese and then translated by Kwang-Chih Chang in 1980 (An 1979, 1980) and an original research article about *Dali man* written by Xinzhi Wu in 1981(Wu 1981) were the earliest publications in WCJs and ushered in the era of Chinese scholars appearing on the international stage. Since then, more Chinese scholars have published articles in WCJs but overall in low quantities, as show in Fig. 5b. This situation has not changed significantly until the start of the twenty-first century; the number of articles has increased dramatically, reaching 284 papers in 2020, though the number decreases slightly in 2021 (*n* = 214). Overall this upward trend will continue in the future.

#### Chinese archaeological research and international academic frontiers

**Fig. 6:**
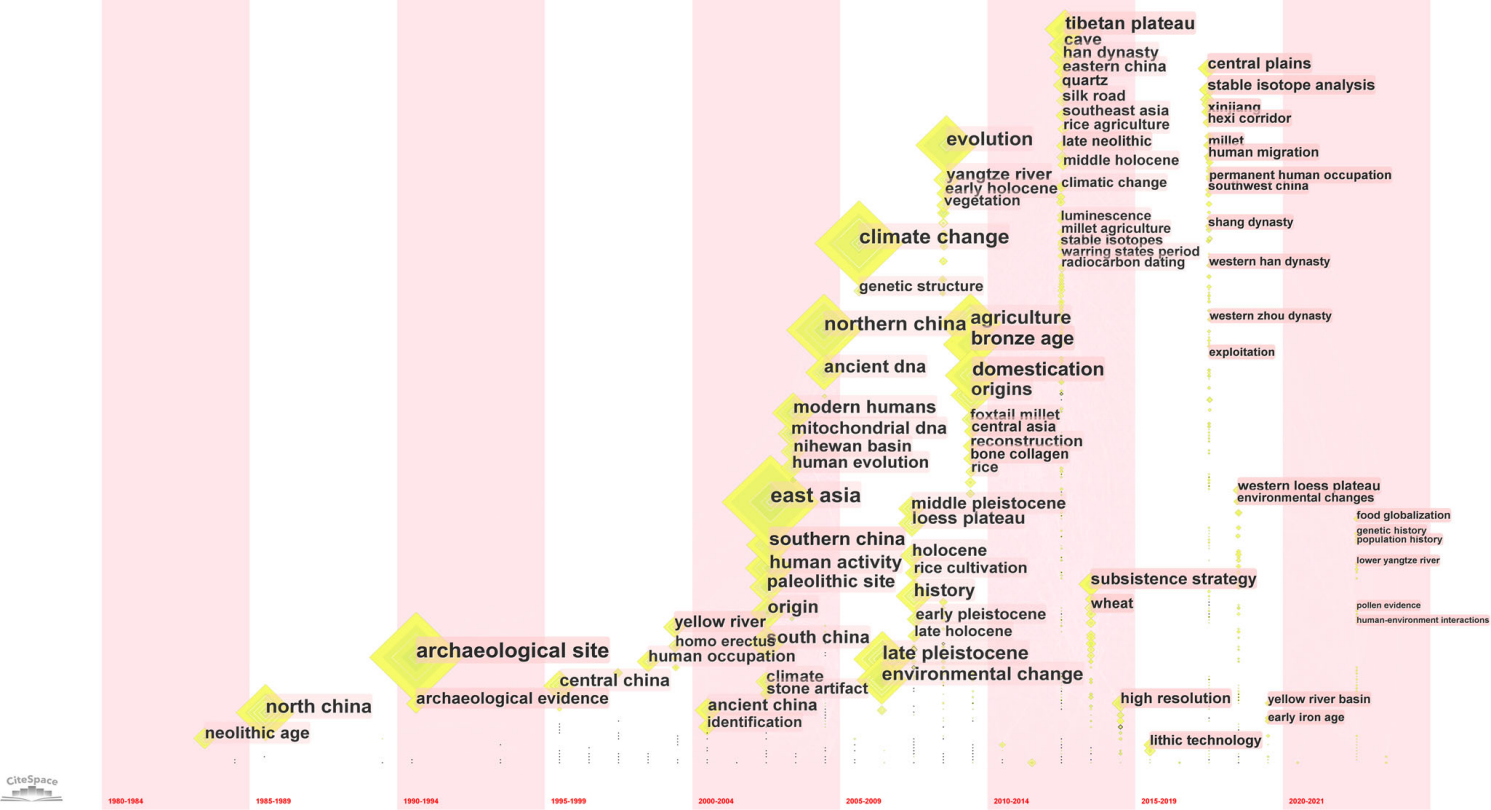
Time-Zone view of high-frequency keywords of articles published by Chinese scholars in WCJs (Created by CiteSpace). The image displays the evolution of keywords over time, from 1980 to 2021. The size of the nodes corresponds to the occurrence frequency of these keywords.

The analysis of keywords’ evolution in different stages was conducted by using the software CiteSpace, providing the results shown in Fig. 6. The nodes and typefaces’ size represent the keywords’ weight. From left to right, this image shows the change of keywords from 1980 to 2021, and the year in which the keywords are located is the average year of their appearance.

Figure 6 indicated that before 2000 the number of keywords was small but the occurrence frequency was high. The number of keywords increased dramatically after 2000, especially from 2002 to 2010; many top 20 high-frequency keywords appeared in this period. Some new high-frequency keywords have emerged since 2018, though the weight is not so important since it just started.

The first high-frequency occurring keyword was “neolithic age”. The keyword that appeared most frequently before 2000 was “north china”, followed by “central china”. These two areas as well as “yellow river” actually represented the core area of the earlier culture and civilization of China. Other keywords including “homo erectus” and “human occupation” were also frequently present as research themes, which focuses on early humans. During 2000–2009 a vast number of new keywords appeared, including some particularly noticeable ones, like “climate change”, “evolution”, “agriculture”, “bronze age”, “domestication”, “origins”, “late pleistocene”, “environmental change” and “ancient dna”. In addition to early humans and their cultures, some topics such as the environment and subsistence strategies became the focus of attention. In terms of the periods, the Bronze Age gained more attention; in terms of method, ancient DNA appeared and was extensively applied for exploring the origin and evolution of prehistoric populations and animals. Some new keywords can be found after 2010: in addition to the core areas like north and central China, some remote frontier regions in the northwest and southwest China have received more and more attention, including “tibetan plateau,” “xinjiang,” “silk road,” “western loess plateau”; in parallel to the prehistorical periods, early Iron Age and historical periods (like “warring states period”, “han dynasty”, “tang dynasty”) began to receive more attention; many new methods of natural sciences including “stable isotopes”, “luminescence”, “starch grains”, “radiocarbon dating” were applied to explore more profoundly the scientific questions. Since 2020 some new keywords have occurred, like “food globalization”, “genetic history”, “population history” and “human-environment interactions”, which represent to some extent the current research frontiers.

The top 20 keywords in articles published in WCJs by Chinese scholars (left) and World scholars (right) in terms of frequency of occurrence are generated by the software CiteSpace, as shown in Table 4. Eight of these keywords were shared, including “archaeological site”, “climate change”, “origin(s)”, “evolution”, “late pleistocene”, “site”, “ancient dna” and “history”. In addition, some of the keywords in the left and right columns look different, but they are all closely related to specific field, such as “environmental change” (left) and “climate” “vegetation” (right), “human activity” “modern humans” (left) and “population” (right). These results suggested that Chinese scholars were highly coupled with hotspots in international academic communities.

**Table 4:** Top 20 high-frequency keywords in articles published in WCJs by Chinese scholars (left) and world scholars (right).

Keywords in articles published by Chinese scholars	Number of occurrences	Keywords in articles published by world scholars	Number of occurrences
East Asia	216	Archaeological site	1790
Archaeological site	210	Evolution	1375
Climate change	199	Origin(s)	1276
Origin(s)	190	History	1192
Northern China	171	Age	928
Agriculture	144	Site	891
North China	142	Climate	632
Evolution	140	Identification	626
Late Pleistocene	137	Patterns	621
Site	137	Record	609
China	133	Climate change	562
Bronze age	128	Holocene	558
Domestication	127	Calibration	554
Environmental change	120	Europe	540
Human activity	115	Late Pleistocene	521
Southern China	113	Ancient DNA	517
Modern humans	104	Diversity	497
Tibetan plateau	97	Vegetation	497
Ancient DNA	96	Population	494
History	93	Bone	485

#### Chinese archaeologists in international academic community

**Fig. 7:**
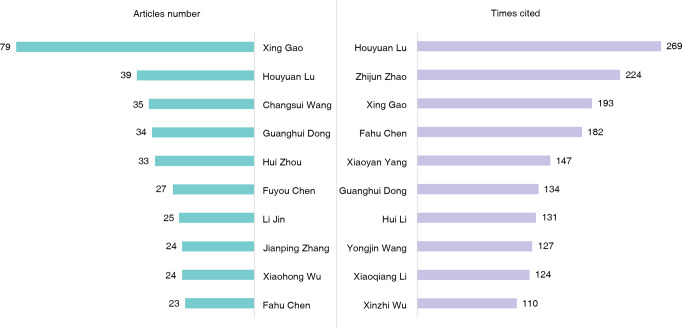
Top 10 high-output and highly cited authors (Created by CiteSpace). The top ten authors with the highest number of published articles are shown on the left, and the most cited authors and the times cited are shown on the right.

High-impact scholars include not only the founders of disciplines, schools or classic theories in a field, but also researchers who are currently active in the frontline of scientific research (Qiu and Ma 2006). The statistical analysis of high output and highly cited authors can help us to understand the core scholars in a field and the trend of research practice in a discipline (Hu 2018). So with the software CiteSpace we analyzed articles number and citation frequency of Chinese scholars in WCJs in order to see the influence of Chinese archaeologists in international academic community. The results are shown in Fig. 7.

It is indicated that the scholar who published the largest number of articles (*n* = 79) is Xing Gao, Paleolithic researcher in Institute of Vertebrate Paleontology and Paleoanthropology (IVPP), Chinese Academy of Sciences (CAS); he is also among the top three highly-cited authors (193 times). Other authors with large number of articles include Houyuan Lu, Changsui Wang, Guanghui Dong, Hui Zhou, and so on. The most frequently cited scholar is Houyuan Lu (269 times), researcher of environmental archaeology in CAS, who is also the second highest output author with 39 papers. The second highly-cited author is Zhijun Zhao (224 times), researcher of botanical archaeology in IA CASS. Other highly cited scholars, include Fahu Chen and Guanghui Dong, who are also among the top ten high output authors.

**Fig. 8:**
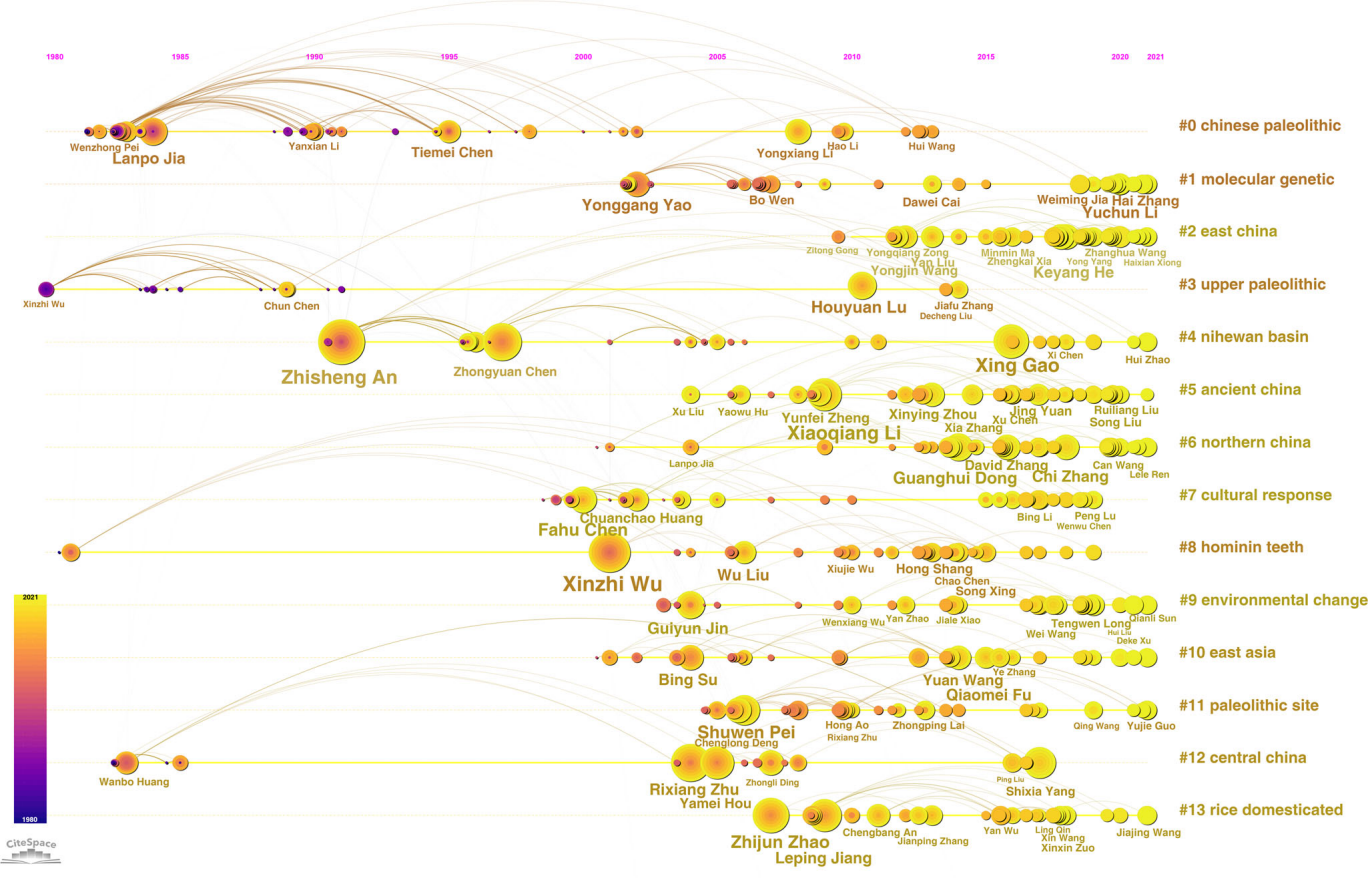
Chronological Changes in Highly Cited Chinese archaeologists (Created by CiteSpace). The biggest 14 clusters produced by the cited authors and their articles’ keywords are shown in the figure and represented by each horizontal timeline, and all clusters are listed vertically in order of size from the largest to the smallest. The horizontal timeline represents a specific keyword cluster and displays the internal connection between authors and keywords across time. All clusters are displayed from left to right, and the cited authors in each cluster are presented on the timeline when they first appeared. The size of nodes represents the weight of the cited frequency.

In order to better understand the evolution of high-impact Chinese archaeologists, we used CiteSpace to generate a timeline view of cited authors, as shown in Fig. 8. According to the clustering algorithm of CiteSpace, the cited authors are divided into 24 clusters and the biggest 14 clusters are indicated in Fig. 8. Each horizontal timeline represents a specific keyword and displays the internal connection between authors and keywords across time. All clusters are displayed from left to right, and the cited authors in each cluster are presented on the timeline when they first appeared. The legend of time for publication is shown on the top and all clusters are listed vertically in order of size from largest to smallest. The nodes are shaped like an annual ring, each ring representing a cited article, and the larger the ring is, the more times it is cited. Different colors represent different publication time, as shown by the bar legend in the lower left corner.

It is indicated by Fig. 8 that the biggest cluster is “#0 chinese paleolithic”. Some clusters like “#3 upper paleolithic”, “#11 paleolithic site”, as well as “#4 nihewan basin” are closely related, so we put them together for analysis. These clusters display some influential authors; Xinzhi Wu is the earliest one, followed by others in the same period, including Lanpo Jia, Wenzhong Pei, and Chun Chen; Zhisheng An is the highly cited author during the 1980s; the foremost representative scholars of the 1990s are Tiemei Chen and Zhongyuan Chen. Many influential scholars appreared after 2000, such as Xing Gao, who published the largest number of articles in WCJs and had very high citation frequency, as well as Houyuan Lu, Yongxiang Li, Shuwen Pei, and so on. The second biggest cluster is “#1 molecular genetic”. Yonggang Yao was a representative scholar in the early stage, and the vast majority of influential scholars in this field appeared after 2018, including Yuchun Li, Hai Zhang, Weiming Jia, etc. The third biggest cluster is “#2 east china”, a geographic area that has been intensively studied since 2010, and many highly-cited scholars have emerged after 2017, such as Keyang He, Yongjin Wang, etc. And two of the earliest studied themes are “#12 central china” and “#8 hominin teeth”; Wanbo Huang was the influential scholar in the early stage during the 1980s and Xinzhi Wu was influential around the year 2000; Rixiang Zhu, Yamei Hou and Wu Liu were highly-cited authors from 2000 to 2005. Other clusters (#5,6,7,9,10,13) are mostly concerntrated after 2005; the highly-cited authors include Fahu Chen, Guanghui Dong, Chi Zhang, Bing Su, Qiaomei Fu, Zhijun Zhao, Leping Jiang, etc.

### Collaboration level of Chinese archaeology in the international academic community

In this era of globalization, collaboration has become a primary way to combine and organize resources into a superior platform for research. The increase in joint international papers signifies an increased scientific collaboration across national boundaries and offers an important area for bibliometric exploration (Luukkonen et al. 1992; Price 1963; Zhang et al. 2020). Here, we analyzed the role of Chinese and foreign experts in WCJs and the distribution of collaborating institutions.

#### Role of Chinese scholars in articles published in WCJs

**Fig. 9:**
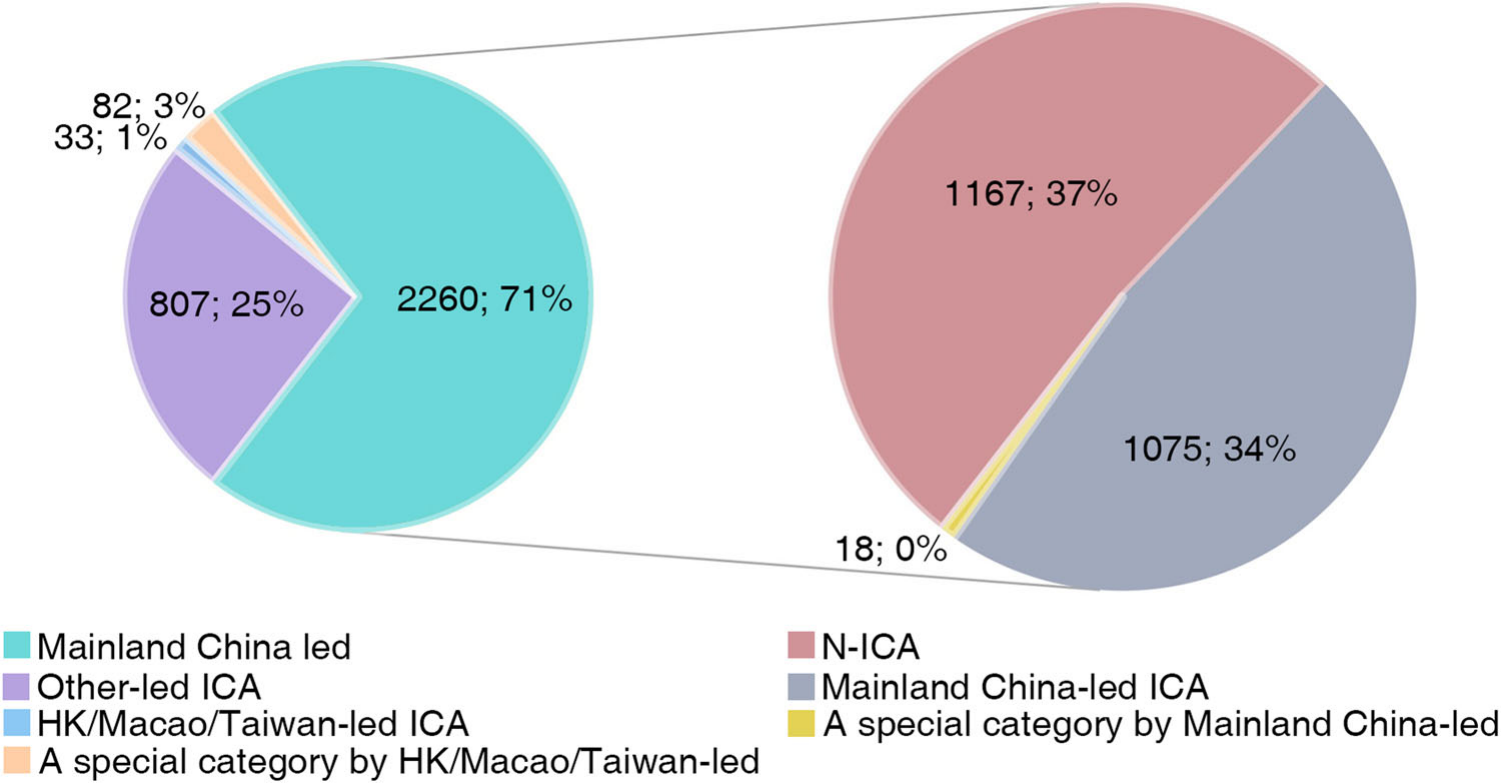
Proportion of different types of collaboration. The pie chart on the left represents four types of international collaboration, and the category “Mainland China led” can be subdivided into three groups, the proportions of which are shown by the pie chart on the right.

The analysis of collaboration type for Chinese scholars’ archaeological papers published in WCJs helps clarify the role of international collaboration in Chinese archaeology. We filtered a total of 3182 papers with Chinese authors, as shown in Figs. 2 and 9. And ICA (*n* = 1915) is the most popular collaboration type, accounting for 60.2% of all papers with Chinese authors, followed by N-ICA (*n* = 1167) accounting for 36.7% of the total, and then by a special category (*n* = 100) with at least one author from Mainland China and other authors from Hong Kong/Macao/Taiwan, accounting for 3.1% of the total. Many articles thus appear to involve cross-regional collaboration.

In terms of the role of Mainland Chinese scholars, 2260 articles were Mainland China-led, accounting for 71% of the total, including 1167 N-ICA articles, 1075 ICA of Mainland China-led articles, and 18 articles involving collaboration with scholars from Hong Kong, Macao, and Taiwan. Another 807 articles were published with foreign scholars as first or corresponding authors, accounting for 25% of the total, and 107 articles were published with scholars from Hong Kong/Macao/Taiwan as the first or corresponding author, accounting for 4% of the total. It thus appears that the majority of archaeological papers with Chinese authors published in WCJs were led by Mainland Chinese scholars.

#### Levels of international collaboration among Mainland Chinese institutions

**Fig. 10:**
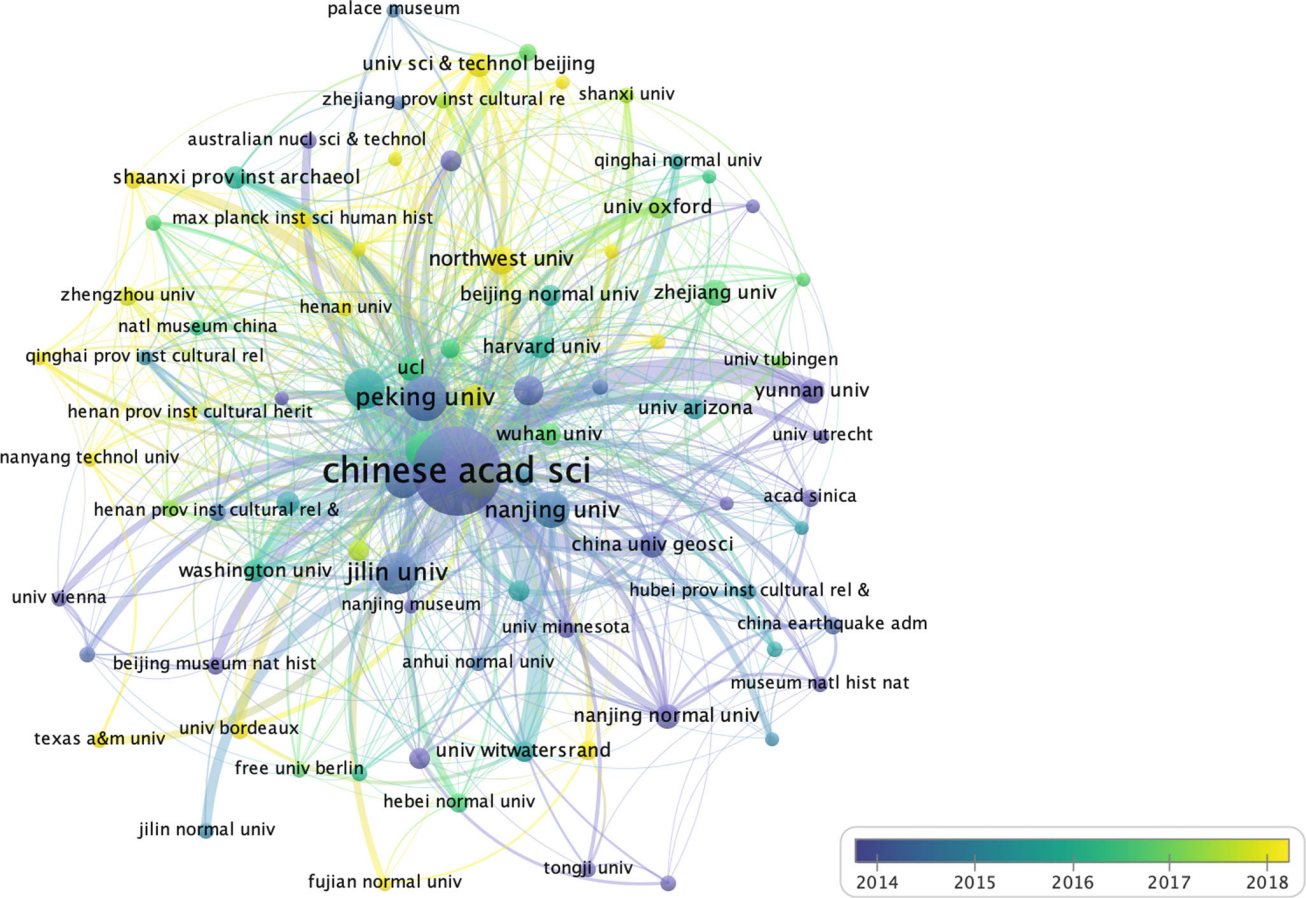
Overlay visualization of institutional collaboration based on publications involving Mainland Chinese researchers in WCJs (Made by VOSviewer). The size of the nodes reflects the number of articles, and the color corresponds to different years of publication by average. The thickness of the links indicates the extent of collaboration between them, and the distance suggests the proximity or distance of academic relationships between organizations.

An examination of institutional collaboration is depicted in Fig. 10. Using VOSviewer, a total of 1494 “organizations” were extracted. When the “minimum number of documents of an organization” and “minimum number of citations of an organization” thresholds were set to 10, the software provided 89 organizations and 782 links. In other words, 89 institutions with document numbers and citation frequencies greater than 10 have collaborated 782 times; of these, 62 were Chinese institutions and 27 were international. The size of the nodes in Fig. 10 reflects the number of publications; the linkage between the nodes reflects the existence of collaboration between them: the thicker the linkage, the closer collaboration; the distance reflects, to some extent, the proximity or distance of the academic relationship between organizations; the different color corresponds to different year of publication by average. An early average publication year might suggest that it started early and lasted for several years, or that it started early and the majority of articles were published at that time; conversely, a later average publication year might imply that it started later or that the majority of articles were published at a later time. Table 5 lists the top 20 domestic and international institutions based on the number of articles published in WCJs.

**Table 5:** Top 20 Chinese (left) and foreign (right) collaborating institutions based on the number of articles published in WCJs.

Chinese Institution	Documents	Average Publication Year	Foreign Institution	Documents	Average Publication Year	Country
Chinese Acad Science	822	2014	University College London	49	2017	England
Peking University	168	2014	Washington University	34	2016	USA
Jilin University	132	2014	Harvard University	32	2016	USA
Chinese Acad Social Science	124	2016	University of the Witwatersrand	28	2016	South Africa
Lanzhou University	107	2016	University of Hawaii Manoa	26	2015	USA
Fudan University	96	2014	University of Oxford	26	2017	England
Nanjing University	96	2015	University of Arizona	24	2015	USA
Shandong University	74	2018	Max Planck Institute for The Science of Human History	22	2019	Germany
University of Science and Technology of China	63	2014	University of Queensland	19	2015	France
Northwest University	52	2019	University of Minnesota	17	2012	USA
China University of Geosciences	48	2014	University of Bordeaux	16	2019	France
Zhejiang University	45	2017	UNESCO	16	2018	France
Nanjing Normal University	41	2013	Australian National University	15	2014	Australia
Sichuan University	37	2018	CNRS	14	2015	France
University of Science and Technology Beijing	37	2018	University of Cambridge	14	2017	USA
Yunnan University	36	2014	University of Vienna	13	2012	Australia
Wuhan University	32	2017	Australian Nuclear Science & Technology Organization	12	2014	Australia
Shaanxi Provincial Institute of Archaeology	31	2016	Texas A&M University	12	2018	USA
Sun Yat-sen University	30	2015	Free University of Berlin	11	2017	Germany
Beijing Normal University	28	2016	University of Nottingham	11	2018	England

As shown in Fig. 10, CAS——especially IVPP——is associated with a large number of publications in WCJs. It is clearly in the center of the collaboration network involving Mainland Chinese scholars, indicating that it has collaborated with many institutions, including Peking University, Jilin University, Fudan University, the China University of Geosciences, and Yunnan University——they were the earliest institutes to establish and have the most frequent international cooperation. These institutions also published the largest number of papers in WCJs. Northwest University and the University of Science and Technology Beijing appeared relatively later; although the average publication years are 2019 and 2018, the collaboration frequency and publication numbers are still very important. And their collaboration with Lanzhou University, Nanjing University, and CAS is also very frequent. In addition to collaboration between domestic institutions, the IVPP also frequently cooperates with foreign institutions. The collaboration with the University of Minnesota and the University of Vienna may start very early, though the document number is not very important. The most frequently-collaborating foreign institutions are the University College London, Washington University, Harvard University, and the University of the Witwatersrand.

Numerous partnerships have been established with foreign institutions of various countries in North America, Europe, and Asia. Collaboration with institutions in the United States is the most common among them. When combined with Table 5, it can be observed that among the top 20 foreign institutions there are seven, nearly half, from the United States, including Harvard University, University of Washington, University of Arizona, and University of Minnesota, among others. Collaboration with institutions in European nations, notably the United Kingdom, France, and Germany, as well as with South Africa, is also important in number. A stable partnership can also be seen with other countries, including Japan, Singapore, India, Israel, and countries in Central and Southeast Asia.

### Characteristics of the journals publishing archaeological papers

WCJs are among the most important indicators for observing the internationalization of Mainland China archaeology because their published articles are important external manifestations of internationalization. International journals are also crucial for making Chinese researchers visible worldwide and obtaining the best resources and quality standards (Zhang et al. 2020). This section investigates the internationalization of Mainland China’s archaeological research through WCJs, including examining how journals are distributed among disciplines, countries, journal quartiles, and collaboration types, with reference to papers published by researchers from Mainland China.

#### Disciplinary distribution of WCJs publishing archaeological articles

**Fig. 11:**
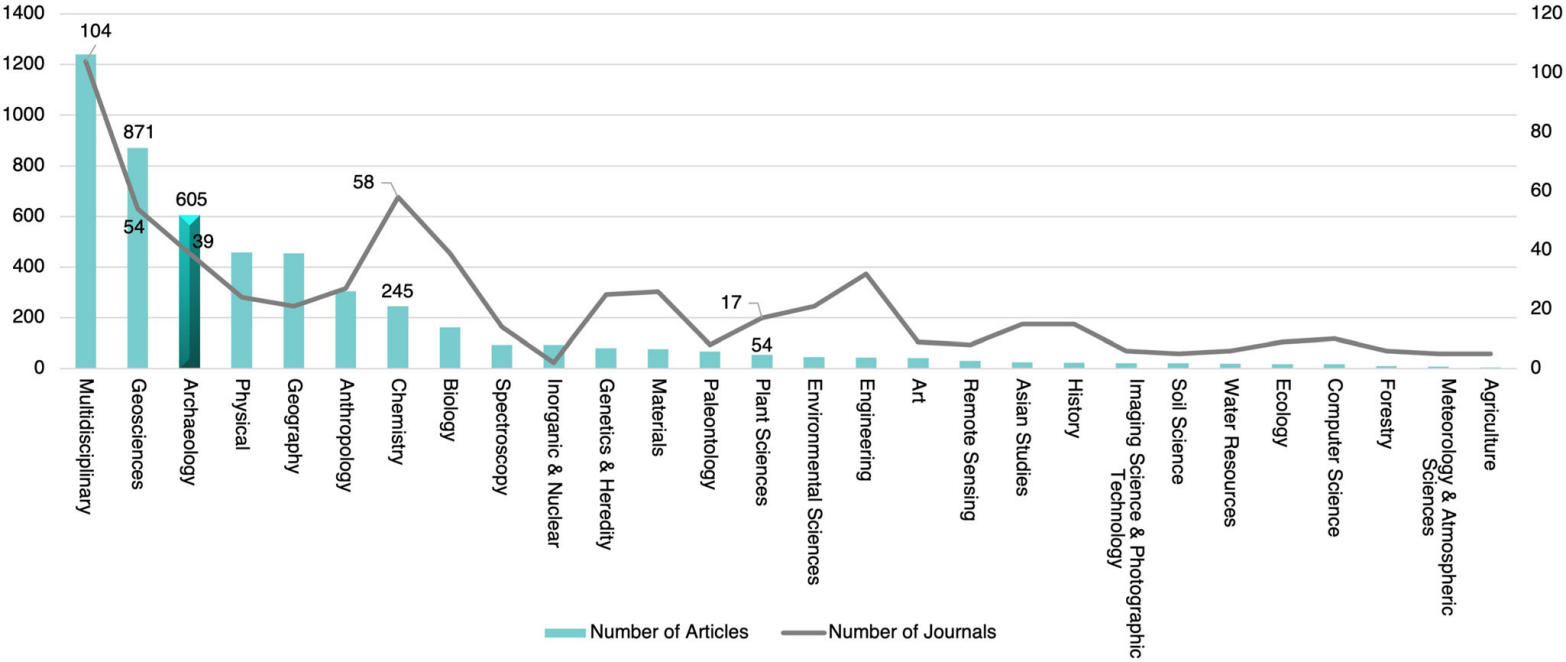
Distribution of article numbers among disciplines to which the journals publishing archaeological articles of Mainland Chinese scholars belong. The number of journals in related disciplines is represented by the folded line, while the quantity of articles corresponding to those journals is indicated by the bars, highlighting the bars of Archaeology.

Archaeology is a profession that benefits from interdisciplinary collaboration, so the statistics on disciplines to which the journals belong can reveal how different disciplines have contributed to internationalization in the field. In total, Chinese archaeologists have published 2,260 archaeology-related papers in 395 international journals belonging to 57 different WoS Category (WC for short). Figure 11 depicts the WC distribution of journals and essays (it only shows WCs with more than five articles). It is indicated that journals belonging to many different disciplines contain archaeology-related papers. The JCR contains 151 journals whose WC is “Archaeology”, in which only 39 “Archaeological” journals *sensu stricto* have published articles (*n* = 605) written by Mainland Chinese scholars.

Multidisciplinary journals are the most popular among Mainland Chinese archaeologists (1241 papers in 104 journals), followed by 54 journals belonging to geosciences with 871 papers. The number of journals and articles in physics and geography are slightly smaller than in archaeology. The articles in anthropology and chemistry are also large in number but slightly less than in archaeology; in terms of the number of journals, the journals belonging to chemistry are ranked second. Generally, except for a small number of journals belonging to archaeology, anthropology, history and art, most WCJs in which Chinese archaeologists published articles belong to the natural sciences.

**Fig. 12:**
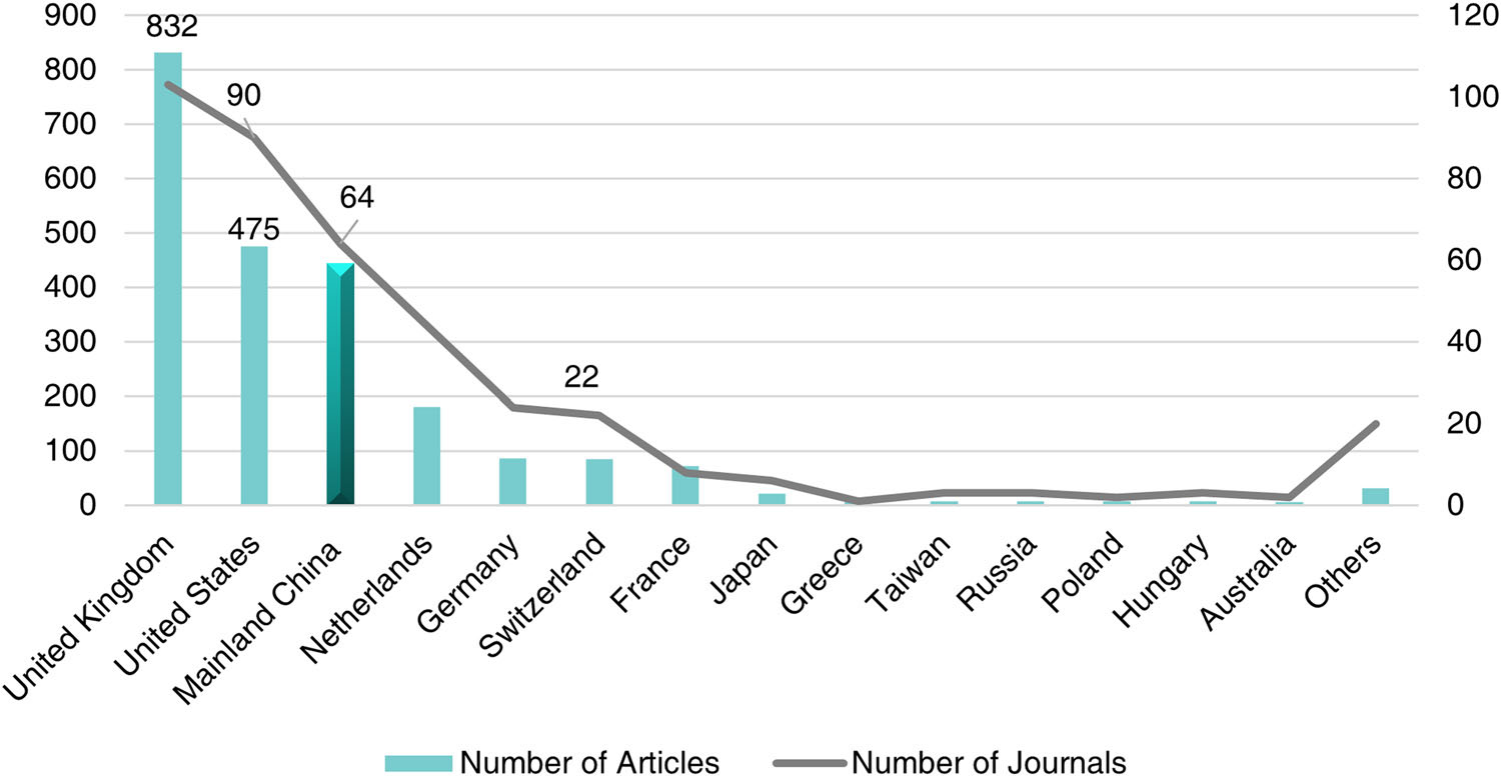
Distribution of numbers of articles published by Chinese scholars among the host countries of the journals in which the articles were published. The number of journals in host countries is represented by the gray folded line, and the quantity of articles corresponding to those journals is indictated by the bars, highlighting the bars of Mainland China.

The distribution of the journal host countries is shown in Fig. 12. In the total of 395 journals, those established by the United States and the United Kingdom are predominant: Chinese archaeologists have published 832 papers in 103 journals from the United Kingdom and 475 articles in 90 journals from the United States. Mainland China-published journals (*n* = 64) contained 444 papers published by Mainland Chinese archaeologists. Among the international journals published by Mainland Chinese institutions, *Chinese Archaeology* published the largest number of articles, and it is the only journal of archaeology *sensu stricto* published internationally by Mainland China and included in the WoS core collection (ESCI index) (see Supplementary Table S3 online for detailed information about the WCJs established by Mainland China’s institutions).

#### Journal quartile distribution

**Fig. 13:**
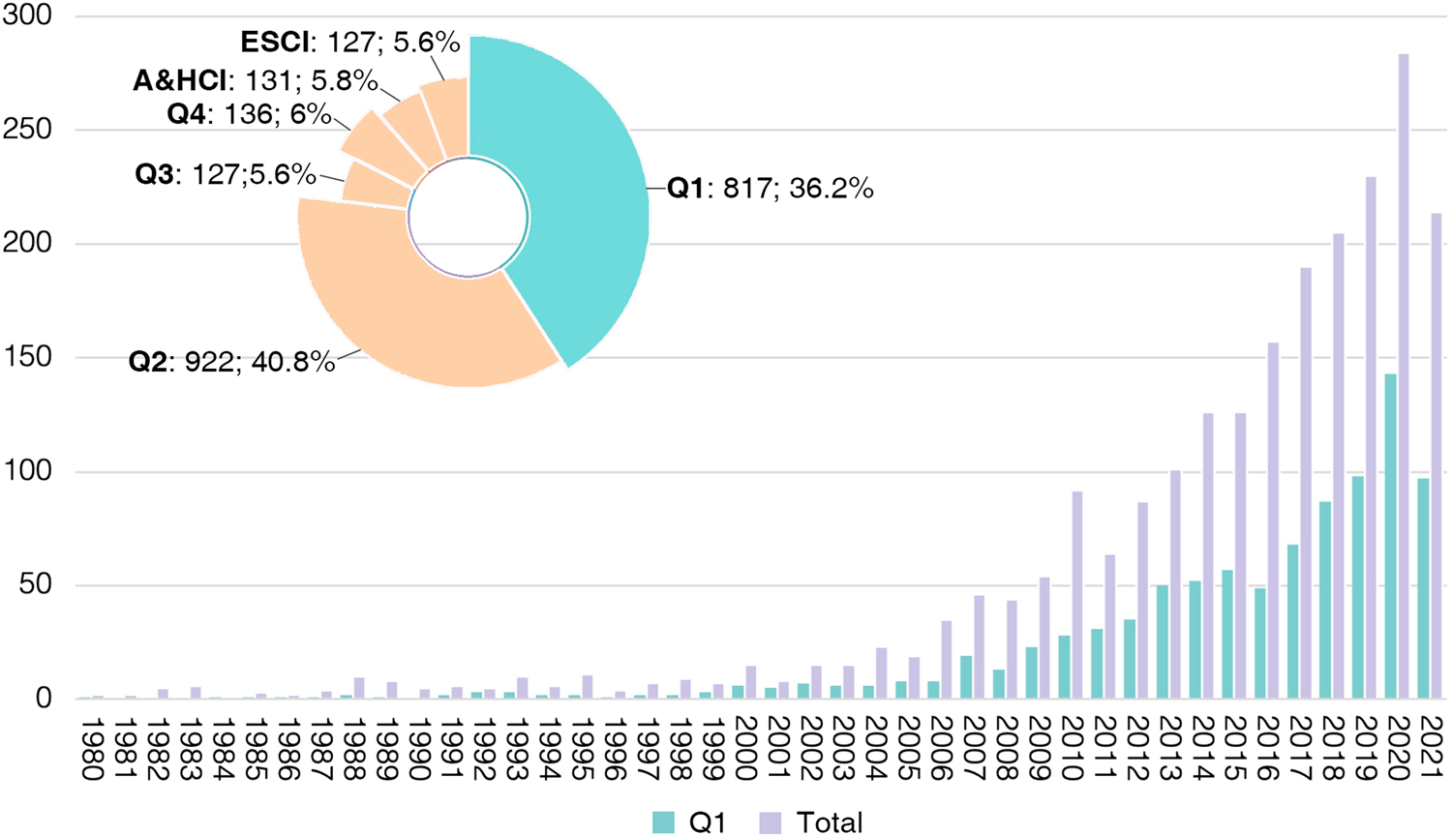
Proportion of different JIF quartiles for the archaeology-related articles published by Chinese scholars in WCJs and diachronic change of article numbers for Q1 and total journals. The proportion of different JIF quartiles is shown on the pie chart on the top left. The bar chart shows the diachronic change of article numbers for Q1 and total journals.

The JIF is a standard indicator of a journal’s impact within and beyond its discipline, and generally speaking, the journals with higher impact factors are considered more prestigious (Garfield 2006). However, the JIF and its use are widely debated at present. We take the view that impact factor is not a perfect indicator of the scientific impact of journals, but it can still be considered as “a gauge of relative quality as judged by both researchers and practitioners” (Saha et al. 2003). The JIF quartiles of WCJs in which Mainland China’s scholars published papers were therefore counted and compared to investigate the different collaboration types and clarify the quality and influence of Mainland Chinese scholars publishing internationally. Figure 13 depicts the proportion of JIF quartiles for international journals in which Mainland Chinese researchers have published articles and the diachronic change of the number of articles published in Q1. We can see the increase in both the total number of international journals and the number of Q1 international journals over the last four decades. Again, most papers were published in Q1 and Q2 international journals, accounting for 40% and 36% of the total articles.

**Fig. 14:**
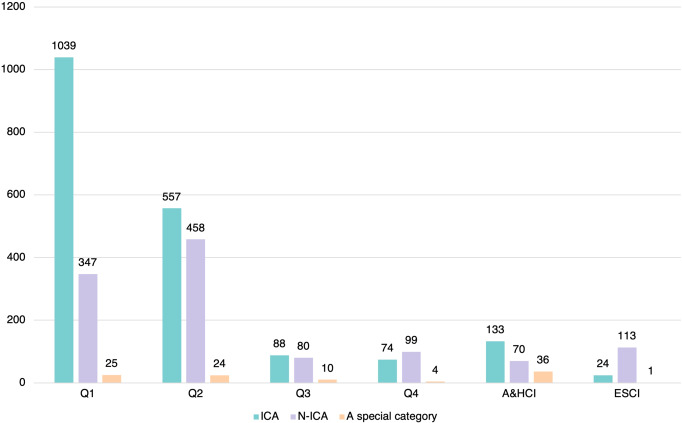
Distribution of article numbers by JIF quartile among different collaboration types. Each cluster of bars represents the different JIF quartiles and includes three different cooperation types.

Figure 14 illustrates the distribution of article numbers by JIF quartiles to different types of international collaboration. The majority of articles in ICA were published in Q1 journals (1039 articles), followed by Q2 journals (557 articles), with the other types of journals being very small in number. In N-ICA, most articles were published in Q2 journals, with 458 articles, followed by Q1 with 347 articles, and ESCI with 113 articles, the majority of which were published in *Chinese Archaeology*. In other words, the number of papers related to ICA and published in high-level international journals is significantly higher than those in N-ICA. In addition, the articles involving collaboration with scholars from Hong Kong, Macao, or Taiwan are much smaller in number than those in the other two collaboration types.

## Conclusion

### Knowledge level of Chinese archaeologists regarding the international archaeological community

The wave of translations of Soviet literature that began in 1950 was a “honeymoon period” for Sino-Soviet archaeology, spurred by a political call to “learn from the advanced experience of the Soviet Union” (Liu and Zhang 2016); 76 papers on Soviet archaeology in Table 3 were mostly translated at that time. Many studies on the archaeology of other communist countries were also translated, including those from Ukraine and Romania (Boriusov and Xiao 1961a, b; Kiselev et al. 1959). During this time, the journal *Archaeology* (then called *Archaeological Newsletter*) became an important venue for bringing in research results related to foreign archaeology. Through numerous translations, Chinese archaeology in the 1950s learned a proletarian archaeological system from Soviet-dominated countries; this had a direct, lasting impact on the historiographic and ethnographic orientation of Chinese archaeology at the time, as well as on archaeological culture and ethnic relations (Liu and Zhang 2016; Zhang 2011). After the early 1960s, translations of Soviet literature were no longer published, and the study of the Soviet Union, which had lasted for more than a decade, came to an abrupt end. Almost no translations were published in Chinese journals for about two decades. As a result, Chinese archaeology began to embark on a relatively independent path of development (Tong 1996).

Since the “Reform and Opening-up” in 1978, translations of foreign archaeology have gradually recovered. *Agricultural Archaeology* released several translations of papers written by international researchers to study agriculture’s roots in China and surrounding nations (Chang et al. 1984; He and Ma 1984). During this time, the examination of the origins of the Chinese state and civilization were the focus of scholarly research in China. The frequent interactions between China and the United States from the 1980s brought to China the New Archaeology, which had originated in the United States after the 1960s (Binford and Chen 1992; Joukowsky and Xu 1988; Watson and He 1992). *Southeastern Culture* and *Cultural Relics in Southern China* became the most important venues for introducing and translating the theories and methods of New Archaeology, with Chinese scholars who had studied abroad playing an essential role. However, there was still a “natural shield” at the time, possibly due to Chinese archaeology’s inherent cultural traditions and research tendencies (Chen 2011). It is undeniable that this wave resulted in a heated discussion about Chinese archaeological theories and methods (Chang 1995; Chen 1997; Rong 1990). Consequently, new techniques and methods related to the paleoenvironment, zooarchaeology, and archaeobotany began to be applied in Chinese archaeology and gradually accepted by more and more Chinese scholars. Since 2000, translations have no longer been the most significant means for acquiring foreign information in China, as more and more Chinese scholars and students have gone abroad for study and exchange.

Through the analysis of translated articles over the years, we found that China has followed a circuitous path of learning from the West: a comprehensive study of the Soviet Union during the 1950s to mutual criticism following the breakdown of Sino-Soviet relations in the early 1960s (Liu and Zhang 2016); relative closure in the 1960s and 1970s to an open, inclusive, and blossoming academic communication landscape today. Generally, Chinese archaeologists have become much better informed about the international archaeological community in multiple ways. However, translating classic articles remain a convenient and effective way for Chinese archaeologists to learn about worldwide academic advances.

### The internationalization of Chinese archaeology

The rapid increase in the number of archaeological articles published in WCJs by Chinese researchers since the 1980s speaks well of significant progress in the internationalization of the field. These AFAs also saw a significant increase in number, especially since 2013 when the “Belt and Road” initiative was launched. However, the AFAs published by Mainland Chinese scholars are still very few, only 1.24% of all archaeological papers in CCJs and WCJs. They account for only 1.2% of all archaeological articles in CCJs and only 4.6% of all archaeological articles published by Mainland Chinese scholars in WCJs. AFAs related to joint Sino-foreign archaeological projects (*n* = 49) have also mostly been published in CCJs (45 articles). Despite the significant increase in the number of articles published by Chinese scholars in WCJs and the number of AFAs by Chinese scholars, Chinese archaeologists still face enormous challenges in gaining full access to the international academic discourse (Chang 1999).

The high-frequency keywords analysis of the articles published by Mainland Chinese scholars in WCJs and comparison with those published by archaeologists across the world indicated that nearly half of the top keywords were shared and included keywords such as “climate change”, “late pleistocene” and “ancient dna”. Although the orientation and review requirements of WCJs have a certain degree of influence on the publication of articles, it can be inferred that Chinese scholars have a strong ability to track hot spots and frontiers in the international archaeological community, showing a high degree of internationalization in these research domains. This type of problem-oriented, interdisciplinary, and cross-regional collaboration is expected to continue and enable Chinese archaeology to enhance its visibility and influence in the international academic community. Besides the above-mentioned areas that focus on the early (prehistoric) stages of mankind and are mainly studied in an interdisciplinary manner, Chinese archaeology has also considered many more scientific questions such as the origins of Chinese civilization, the origins of the state, and archaeology of historical periods, which form the topics of most Chinese archaeologists. Actually, our analysis of keywords evolution in different stages suggested a gradual shift in scholarly attention from the prehistoric to the historical period and from central region to remote frontier areas of China. It is a gratifying change, because the progress of research on these topics is also worth being presented to the world, as they can provide irreplaceable models for world archaeology (Chang 1999).

The analysis of influential scholars in different stages revealed that some researchers have made preliminary attempts to the internationalization of Chinese archaeological research since the 1980s, such as Xinzhi Wu, Lanpo Jia, and Wenzhong Pei, who were especially the paleoanthropologists and paleolithic researchers. A large number of scholars have participated in international academic discussion since 2000. And more and more scholars took important part in international publication in recent years. This is remarkable advancement in the internationalization of Chinese archaeology.

In sum, although the overall number of articles published by Chinese archaeologists in WCJs has increased at an unprecedented rate over the last two decades and more and more scholars become active in international publication, it still forms only a small proportion of all articles in WCJs in archaeology and also a drop in the ocean compared to the number of articles published in CCJs. So, more effort may need to be made to enhance the visibility of Chinese archaeology in the international academic community.

### International collaboration

Regarding the role of Mainland Chinese scholars and institutions in publishing articles in WCJs, it was revealed that Mainland Chinese scholars took a leading role in cross-national/regional collaboration, which is a very popular type of collaboration for Chinese scholars publishing articles in WCJs. In all archaeological articles with Chinese authors (*n* = 3182), most (*n* = 2015) involved cross-regional collaboration with other countries or Hong Kong/Macao/Taiwan (i.e., ICA), followed by articles without collaboration (i.e., N-ICA, *n* = 1167). While detailed analysis suggests that the N-ICA and Mainland China-led articles together accounted for about 71% of the total number of articles (*n* = 2260), only a small proportion of articles (*n* = 922) can be attributed to ICA other-led (involving collaboration with other countries or Hong Kong/Macao/Taiwan). It can be inferred that a significant number of Chinese scholars are fully capable of designing, completing, and publishing research independently on issues key to the international research community and are increasingly prominent as major contributors in collaborative research with other countries or regions. This may result from policies related to preference and monetary incentives because most institutions’ scientific research evaluation system sees the role of “first author” or “corresponding author” as a condition for recognizing achievement (Quan et al. 2017), which may have contributed significantly to the increase in the number of N-ICA and Mainland China-led ICA articles.

Numerous international institutions (*n* > 27) have collaborated with Chinese archaeological institutions, including organizations in the United States, Europe, Asia, Africa, Near East, and Asia. Among these institutions, University College London has the highest number of publications, but the United States has the highest total number of institutions. Chinese students and scholars who visit the UK and US and then return home play a vital role in promoting cooperation between China and English-speaking countries (Jin et al. 2007). Furthermore, owing to geopolitical ties and historical, linguistic, and cultural similarities, there is much cooperation between mainland China and neighboring Asian countries, thus significantly enhancing the depth and breadth of international cooperation in archaeological research in mainland China. At least 62 archaeological institutions in China have collaborated internationally, and these institutions cover almost all archaeology-related institutes and universities in China, with CAS taking the absolute leading role, followed by Peking University, Jilin University, CASS, and Lanzhou University. The depth and breadth of international collaboration in Chinese archaeology are considerable, but clearly unbalanced among Chinese institutions in terms of the number and scale of international collaborations. This may be related both to the high degree of coupling of some institutional research areas with international hotspots, and to the national economic and policy support they have received.

### Journal characteristics

Three characteristics regarding the WCJs publishing archaeological papers were revealed in our analyses.

First, the WCJs in which Chinese scholars have published archaeological articles have a prominent interdisciplinary character, with a particular predominance of journals in the natural sciences. Of the 395 international journals that have published articles on Chinese archaeology, only 39 belong to the category of archaeology *sensu stricto*. Of the remaining journals, apart from 51 journals belonging to anthropology, history, and art, 104 are interdisciplinary within the natural sciences. The natural sciences that intersect with archaeology are very broad, with the most numerous being geosciences, followed by physics, geography, chemistry, and biology. This is closely related to the interdisciplinary character of world archaeology that has emerged over the last four decades, and this trend is set to continue.

Second, the United Kingdom and the United States host almost half (*n* = 193) of all WCJs related to archaeology, arguably dominating the field and reflecting the hegemony of English-language journals. The number of journals (*n* = 64) established by Mainland Chinese institutions represents only 16% of the total. Of these journals, only one——*Chinese Archaeology*—is strictly attributed to archaeology, while all other journals are interdisciplinary, with those attributed to geosciences and geography being the most numerous. The number of archaeological articles published in Chinese journals is almost equal to that of American journals, although there are far fewer Chinese journals, which suggests that China needs to establish more English-language journals, especially strictly related to the field of archaeology, to provide a broader platform for the international community to better understand the archaeological research of Chinese scholars.

Third, there was a definite trend among Chinese archaeologists to publish more articles in high-impact journals, especially involving international collaboration. Most of the archaeological articles published by Mainland Chinese scholars were in Q1 (40% of the total articles) and Q2 journals (36% of the total articles). The number of articles published in Q1 journals has increased dramatically over the last decade, and nearly 60% of articles published in Q1 journals involved collaborations between Chinese and international institutions. This suggests that some Chinese scholars are capable of conducting scientific research and publishing the results independently in high-impact journals, while most scholars require international collaboration to bring their research to the international fore. This trend may be related to the Chinese academic evaluation system, as for a considerable period, the JIF has been an important criterion for assessing scholars’ research results and allocating research funding, and for numerous reasons (including language barriers), research results that include international collaborations are more likely to be accepted by high-impact journals. Finally, it is important to note that only two English-language journals close to archaeology hosted in China are in Q1, with the majority in Q2, Q3, and Q4, which suggests that the international influence of Chinese journals needs to be improved.

## Discussion

The internationalization of Chinese archaeology has increased significantly in the last few decades, especially since the start of the twenty-first century. Multiple factors may continue to drive the international development of Chinese archaeology. Several policies launched by the Chinese government have directly or indirectly contributed to the internationalization of the social sciences and humanities, including Chinese archaeology: “Outline of national cultural reform and development plan in the “12th Five-Year” (CCCPC, GOSC 2011); the “Going out” plan for the humanities and social sciences in universities (MOE 2011); the “Project for Social Science Prosperity in Higher Education Institutions” (MOE, MOF 2011); the Decision of the CPC Central Committee on “Major Issues Pertaining to Deepening Reform of the Cultural System and Promoting the Great Development and Flourishing of Social Culture”(CCCPC 2011); the “National ‘12th Five-Year Plan’ for Philosophy and Social Science Research” (NOPSS, 2011); the “Belt and Road Initiative” (NDRC et al. 2015); the “‘14^th^ Five -Year Plan’ for archaeological work”(NCHA 2022); President Xi’s speech at the symposium on philosophical and social science work (Xi 2016); and the “Building an archaeology with Chinese characteristics, Chinese style and Chinese flair” (Xi, 2020) and the “advancing study of Chinese civilization”(Xi 2022) proposed by President Xi.

Research funding is an important factor for promoting research and the internationalization of Chinese archaeology. For the last two decades, multiple sources of financial support have been available for scholars in Mainland China: National Social Science Fund; National Natural Science Foundation; Fund of the Ministry of Education; Archaeological Survey and Excavation Fund of State Bureau of Cultural Relics at the national level; Provincial Social/Natural Science Fund and Funding for salvage excavations of archaeological sites provided by major construction projects at the local level. Sometimes funding from foreign institutions has also been available for conducting international collaborative research. These funds have played a crucial part in the development of scientific research and knowledge production at both the national and international levels.

The academic evaluation criteria and monetary reward systems have also influenced the internationalization of Chinese archaeology. In China’s publication-oriented academic evaluation system, scholars in archaeology-related institutions are expected to publish their research in high-impact international journals (e.g., SCI-indexed Q1 and Q2 journals, SSCI/A&HCI indexed journals), both for pragmatic purposes and for prestige. This trend is particularly apparent in natural science–led institutions, such as CAS and key institutes and universities where natural sciences and humanities go hand in hand (e.g., “Project 211/985/Double First-Class”–sponsored key universities and CASS). Most institutions also take the first author/corresponding author as a condition for recognizing research achievements, which may have contributed to the significant increase in the number of N-ICA articles and Mainland China-led ICA articles.

Our statistical analyses indicated that the proportion of Chinese archaeologists publishing in WCJs is extremely low. Of the 103,840 archaeology-related articles retrieved from the WoS database, only 3,182 were published by Chinese scholars (3.1% of the total), and Chinese scholars published 14 times more archaeology-related articles in CCJs (*n* = 40,865) than they did in WCJs (*n* = 3,182). Almost all articles on joint Sino-foreign archaeological projects have been published in CCJs in the last decade. From a quantitative perspective, the share of Chinese archaeology in international scholarship is not proportional to the size of the field. In addition, the recent policy of placing a high priority on domestic SSCI-indexed journals (MOE 2020; MOST, 2020) reflects a trend toward localization in Mainland China’s academic policy, which seems to be opposed to disciplinary internationalization. The impact of this policy may vary from person to person. Some researchers (especially in natural sciences–led institutions) who studied abroad and need to maintain their international cooperation networks will likely continue to publish internationally, but with some concerns about the political and financial support for international collaboration and publication (especially after the outbreak of COVID-19). Others who previously focused on domestic research and published mainly in CCJs may not be much affected by this policy. In objective terms, disciplinary internationalization and localization should not be considered as opposed to each other, but as an issue of dynamic balance (Sivertsen 2018). At present, we need more time to observe where the trends of internationalization and localization in Chinese archaeology are heading.

The internationalization of archaeology in Mainland China has increased significantly compared to decades ago, but it has not become a dominant trend. It still faces many challenges, both international and domestic. China has an extremely rich archaeological and cultural heritage combined with a continuous textual record, which is precious in providing a globally unique pattern of civilization evolution (Chang 1989). Chinese archaeology and the international community need each other. It is hoped that Chinese archaeological research will shift from an inward-looking historiographical tradition to an outward-looking one (Chang 1981; Falkenhausen 1993) that will help to “tell the Chinese story, spread the Chinese voice” by increasing China’s soft power internationally (Xi 2013).

The internationalization of scientific research encompasses multiple aspects: policies and projects at the national level to promote international development; international courses; student exchanges; the number of international staff at the institutional level; international cooperation; participation in international conferences and organizations; international publication at the individual level, *etc*. (Zhang et al. 2020). This paper was thus limited in terms of research sources and perspectives, yet the bibliometric method based on individual’s scholarly publication has indeed proven to be very effective and promising to explore certain important issues regarding a discipline. Based on this, new analysis can certainly be conducted in future to discuss more interesting topics, such as gender or institution imbalances in publication trends, preferences for topics of Doctoral and Master’s thesis, the degree of engagement of foreign scholars in Chinese archaeology-related research, the process of acceptance of Chinese archaeological research by foreign scholars and vice versa, the relationship between large archaeological park and interest of academic and public community, and so on, if new data including monographs, dissertations, and papers in non-core journals and detailed information about the gender of authors, the scientific projects at national and institutional level, student exchange, origin and number of international staff and involvement in international conferences and organizations can be fully collected. Therefore, it seems that the flood gates of bibliometric analysis have just been opened in the field of Chinese archaeology. It is expected that more detailed analysis by using this method will bring us new insights and form more comprehensive understanding about the discipline.

## Supplementary information

Bringing in and going abroad-supplementary information

## Data Availability

The raw bibliometric data were collected from Web of Science (Clarivate Analytics) and CNKI. Two official licenses are required to access these two databases, the developers and owners of which regularly charge fees to institutions to grant licenses to researchers within the institutions. The researchers have access to both databases precisely because the institutions pay the fees and obtain the licenses, so the researchers themselves are not allowed to alienate the right to use these two licenses and to disclose all the raw data in a complete way, or they may be considered to be violating the rights of both databases. Therefore, the raw data used in this paper cannot be posted in a repository.
