# Supplementary material for: “Bringing in” and “Going abroad”: A bibliometric evaluation of the internationalization of archaeology in Mainland China
Source: Humanit Soc Sci Commun. 2023 Jun 3;10(1):281. doi: 10.1057/s41599-023-01800-0 (PMC10239220; doi:10.1057/s41599-023-01800-0)
Supplement: Supplementary file 1 — Bringing in and going abroad-supplementary information [file 41599_2023_1800_MOESM1_ESM.docx]

“Bringing in” and “Going abroad”: A Bibliometric Evaluation of the Internationalization of Archaeology in Mainland China

Xuan Wei, Wentai Lou, Ting Li, Ruxi Yang, Yinghua Li

**Supplementary information**

Table S1 Abbreviations used in this article

| **Abbreviations** | **Original words** |
| --- | --- |
| CCJs | Chinese core journals |
| WCJs | World core journals |
| ICAs | International collaboration articles |
| N-ICAs | Non-international collaboration articles |
| AFAs | Articles on foreign archaeology |
| CNKI | China National Knowledge Infrastructure |
| WoS | Web of Science |
| JCR | Journal Citation Reports |
| WC | WoS Category |
| JIF | Journal impact factor |
| CAS | Chinese Academy of Sciences |
| IVPP | Institute of Vertebrate Paleontology and Paleoanthropology |
| CASS | Chinese Academy of Social Sciences |
| BRI | Belt and Road Initiative |

Table S2 List of joint Sino-foreign archaeological projects compiled in this paper

| **Country** | **Site(s)** | **Institution(s)** | **References** |
| --- | --- | --- | --- |
| Cambodia | Chau Say Temple | Chinese Academy of Cultural Heritage | ([Qiao and Li, 2003](#_ENREF_20); [Qiao et al., 2018](#_ENREF_21)) |
|  | Ta Keo | Chinese Academy of Cultural Heritage | ([Wang, 2016](#_ENREF_35); [Yu et al., 2017](#_ENREF_38)) |
| Honduras | Copan Site | CASS | ([Li et al., 2017](#_ENREF_13); [Li, 2018](#_ENREF_12); [Li et al., 2020](#_ENREF_11)) |
| Kazakhstan | Lahate | Shaanxi Academy of Archaeology | ([Ding et al., 2017](#_ENREF_7); [Sun et al., 2020](#_ENREF_30)) |
| Kenya | Gedi Ruins | Peking University | ([Liu et al., 2012](#_ENREF_19)) |
|  | Malindi | Peking University | ([Zhao et al., 2012](#_ENREF_45); [Ding, 2014](#_ENREF_5); [Zhang et al., 2014](#_ENREF_43); [Ding, 2017](#_ENREF_6); [Siu et al., 2020](#_ENREF_28)) |
|  | Mambrui | Peking University | ([Siu et al., 2021](#_ENREF_27)) |
|  | Mombasa Wreck | Peking University | ([Qin et al., 2014](#_ENREF_22)) |
|  | Ungwana Site | Peking University | ([Ding and Qin, 2016](#_ENREF_8)) |
| Kyrgyzstan | Rechka Site | Shaanxi Academy of Archaeology | ([Wang et al., 2020](#_ENREF_36)b) |
| Mongolia | Airagiin Gozgor | Renmin University of China | ([Rong et al., 2021](#_ENREF_25)) |
|  | Gol Mod Cemetery | Henan Provincial Institute of Cultural Relics and Archaeology | ([Zhou et al., 2018](#_ENREF_47); [Zhou et al., 2021](#_ENREF_46)) |
|  | Khulhiin Am Site | Archaeological Institute of Inner Mongolia | ([Tarra et al., 2009](#_ENREF_33); [Cai et al., 2013](#_ENREF_1); [Cai et al., 2016](#_ENREF_2)) |
|  | Khirgisiin Khooloin | Shanxi University; Inner Mongolian Institute of Cultural Relics and Archaeology | ([Chen et al., 2016](#_ENREF_4); [Song et al., 2016](#_ENREF_29)) |
|  | Khermental City Site | Jilin University | ([Li et al., 2018](#_ENREF_10); [Sarrenbillige et al., 2020](#_ENREF_26)) |
| Russia | Kalyvanskoe | Nanjing University | ([Zhang et al., 2017](#_ENREF_40)) |
|  | Maritime Region | CASS | ([Zhao et al., 2005](#_ENREF_44)) |
|  | Troysky | Jilin University | ([Feng and Aerjin, 2006](#_ENREF_9); [Tang et al., 2008](#_ENREF_32); [Zhang et al., 2008](#_ENREF_41), [2009](#_ENREF_42)) |
|  | Primorsky | Jilin Institute of Cultural Relics and Archaeology | ([Liang and Xie, 2016](#_ENREF_14)) |
| Tadzhikistan | Kaskar Cemetery | Northwest University;Luoyang Municipal Institute of Cultural Relics and Archaeology | ([Liang et al., 2020](#_ENREF_17)) |
| Tajikistan & Uzbekistan |  | Northwest University;  National Museum of China;  Shaanxi Academy of Archaeology | ([Ren, 2014](#_ENREF_23), [2015](#_ENREF_24); [Xi, 2019](#_ENREF_37)) |
| Uzbekistan | Mingtepa Site | CASS | ([Zhu et al., 2017](#_ENREF_48); [Zhu and Matbabaev, 2019](#_ENREF_49)) |
|  | Rabat Cemetery | Northwest University | ([Liang et al., 2018](#_ENREF_16); [Liu et al., 2020](#_ENREF_18); [Tang et al., 2020](#_ENREF_31); [Liang et al., 2021](#_ENREF_15)) |
|  | Sazagan Site | Northwest University; Luoyang Municipal Institute of Cultural Relics and Archaeology | ([Yusuf et al., 2018](#_ENREF_39); [Wang et al., 2020](#_ENREF_34)a) |
| Vietnam | central and northern regions of Vietnam | Sichuan Province Institute of Cultural Relics and Archaeology | ([Chen et al., 2021](#_ENREF_3)) |

Note: Articles by scholars from Mainland China about joint Sino-foreign archaeological projects collected in this paper were all from 27 CCJs and WoS core collection, and the few papers published in other journals are not included in this table.

Table S3 List of WCJs established by Mainland China’s institutions

| **Journal** | **Number of publications** | **Indexed in** | **JIF（2021,the best WC）** | **Category** |
| --- | --- | --- | --- | --- |
| CHINESE ARCHAEOLOGY | 96 | ESCI | ESCI | Archaeology |
| CHINESE SCIENCE BULLETIN | 89 | SCIE | Q2 | Multidisciplinary Sciences |
| SCIENCE CHINA-EARTH SCIENCES | 56 | SCIE | Q2 | Geosciences, Multidisciplinary |
| SPECTROSCOPY AND SPECTRAL ANALYSIS | 35 | SCIE | Q4 | Spectroscopy |
| JOURNAL OF GEOGRAPHICAL SCIENCES | 23 | SCIE | Q2 | Geography, Physical |
| SCIENCE BULLETIN | 14 | SCIE | Q1 | Multidisciplinary Sciences |
| SCIENCE IN CHINA SERIES D-EARTH SCIENCES | 9 | SCIE | Q2 | Geosciences, Multidisciplinary |
| SCIENCE IN CHINA SERIES E-TECHNOLOGICAL SCIENCES | 8 | SCIE | Q3 | Materials Science, Engineering, Multidisciplinary |
| ACTA GEOLOGICA SINICA-ENGLISH EDITION | 7 | SCIE | Q4 | Geosciences, Multidisciplinary |
| NATIONAL SCIENCE REVIEW | 5 | SCIE | Q1 | Multidisciplinary Sciences |
| PROGRESS IN NATURAL SCIENCE-MATERIALS INTERNATIONAL | 5 | SCIE | Q3 | Materials Science, Multidisciplinary |
| SCIENCE CHINA-TECHNOLOGICAL SCIENCES | 5 | SCIE | Q1 | Materials Science, Multidisciplinary; Engineering |
| CHINESE SOCIOLOGY AND ANTHROPOLOGY | 4 | SSCI | Q4 | Sociology; Anthropology |
| JOURNAL OF GENETICS AND GENOMICS | 4 | SCIE | Q2 | Biochemistry & Molecular Biology; Genetics & Heredity |
| ARTS OF ASIA | 3 | A&HCI | AHCI | Asian Studies; Art |
| CHINESE GEOGRAPHICAL SCIENCE | 3 | SCIE | Q3 | Environmental Sciences |
| CHINESE JOURNAL OF GEOPHYSICS-CHINESE EDITION | 3 | SCIE | Q4 | Geochemistry & Geophysics |
| CHINESE MEDICAL JOURNAL | 3 | SCIE | Q2 | Medicine, General & Internal |
| FRONTIERS OF EARTH SCIENCE | 3 | SCIE | Q3 | Geosciences, Multidisciplinary |
| INTERNATIONAL JOURNAL OF DIGITAL EARTH | 3 | SCIE | Q1 | Remote Sensing; Geography, Physical |
| JOURNAL OF SYSTEMATICS AND EVOLUTION | 3 | SCIE | Q1 | Plant Sciences |
| PROGRESS IN NATURAL SCIENCE | 3 | SCIE | Q2 | Multidisciplinary Sciences |
| CHEMICAL JOURNAL OF CHINESE UNIVERSITIES-CHINESE | 2 | SCIE | Q4 | Chemistry, Multidisciplinary |
| CHEMICAL RESEARCH IN CHINESE UNIVERSITIES | 2 | SCIE | Q3 | Chemistry, Multidisciplinary |
| CHINESE JOURNAL OF ANALYTICAL CHEMISTRY | 2 | SCIE | Q4 | Chemistry, Analytical |
| CHINESE PHYSICS C | 2 | SCIE | Q2 | Physics, Nuclear; Physics, Particles & Fields |
| JOURNAL OF ARID LAND | 2 | SCIE | Q3 | Environmental Sciences |
| JOURNAL OF MOUNTAIN SCIENCE | 2 | SCIE | Q3 | Environmental Sciences |
| PEDOSPHERE | 2 | SCIE | Q1 | Soil Science |
| SCIENCE CHINA-LIFE SCIENCES | 2 | SCIE | Q1 | Biology |
| SCIENCE IN CHINA SERIES G-PHYSICS MECHANICS & ASTRONOMY | 2 | SCIE | Q2 | Physics, Multidisciplinary |
| ZOOLOGICAL RESEARCH | 2 | SCIE | Q1 | Zoology |
| ACTA BOTANICA SINICA | 1 | SCIE | Q3 | Biochemistry & Molecular Biology; Plant Sciences |
| ACTA GEOLOGICA SINICA | 1 | SCIE | Q4 | Geosciences, Multidisciplinary |
| ACTA GEOPHYSICA SINICA | 1 | SCIE | Q1 | Geosciences, Multidisciplinary |
| ACTA PETROLOGICA SINICA | 1 | SCIE | Q3 | Geology |
| ACTA PHYSICA SINICA | 1 | SCIE | Q3 | Physics, Multidisciplinary |
| ACTA ZOOLOGICA SINICA | 1 | SCIE | Q1 | Zoology |
| APPLIED GEOPHYSICS | 1 | SCIE | Q4 | Geochemistry & Geophysics |
| CELL RESEARCH | 1 | SCIE | Q1 | Cell Biology |
| CHINA GEOLOGY | 1 | ESCI | ESCI | Geology |
| CHINESE JOURNAL OF CHEMICAL ENGINEERING | 1 | SCIE | Q2 | Engineering, Chemical |
| CHINESE JOURNAL OF OCEANOLOGY AND LIMNOLOGY | 1 | SCIE | Q4 | Oceanography; Limnology |
| CHINESE PHYSICS | 1 | SCIE | Q2 | Physics, Multidisciplinary |
| CHINESE STUDIES IN HISTORY | 1 | A&HCI | AHCI | Asian Studies; History |
| CONTEMPORARY CHINESE THOUGHT | 1 | A&HCI | AHCI | Asian Studies; Philosophy |
| FOREIGN LITERATURE STUDIES | 1 | A&HCI | AHCI | Literature |
| FRONTIERS OF AGRICULTURAL SCIENCE AND ENGINEERING | 1 | ESCI | ESCI | Agronomy |
| FRONTIERS OF HISTORY IN CHINA | 1 | ESCI | ESCI | History |
| JOURNAL FOR THE STUDY OF CHRISTIAN CULTURE | 1 | ESCI | ESCI | Religion |
| JOURNAL OF COMPUTER SCIENCE AND TECHNOLOGY | 1 | SCIE | Q3 | Computer Science, Hardware & Architecture, Software Engineering |
| JOURNAL OF EARTH SCIENCE | 1 | SCIE | Q2 | Geosciences, Multidisciplinary |
| JOURNAL OF INFRARED AND MILLIMETER WAVES | 1 | SCIE | Q4 | Optics |
| JOURNAL OF INORGANIC MATERIALS | 1 | SCIE | Q4 | Materials Science, Ceramics |
| JOURNAL OF INTEGRATIVE PLANT BIOLOGY | 1 | SCIE | Q1 | Biochemistry & Molecular Biology; Plant Sciences |
| JOURNAL OF OCEAN UNIVERSITY OF CHINA | 1 | SCIE | Q4 | Oceanography |
| JOURNAL OF ROCK MECHANICS AND GEOTECHNICAL ENGINEERING | 1 | SCIE | Q1 | Engineering, Geological |
| LANDSCAPE ARCHITECTURE FRONTIERS | 1 | ESCI | ESCI | Architecture |
| LASER & OPTOELECTRONICS PROGRESS | 1 | ESCI | ESCI | Optics; Engineering, Electrical & Electronic |
| MOLECULAR PLANT | 1 | SCIE | Q1 | Biochemistry & Molecular Biology; Plant Sciences |
| ROCK AND SOIL MECHANICS | 1 | ESCI | ESCI | Engineering, Geological |
| SCIENCE IN CHINA SERIES B-CHEMISTRY | 1 | SCIE | Q4 | Chemistry, Multidisciplinary |
| SCIENCE IN CHINA SERIES C-LIFE SCIENCES | 1 | SCIE | Q2 | Biology |
| SCIENTIA SINICA | 1 | ESCI | ESCI | Multidisciplinary Sciences |

Reference：

Cai DW, Chen X, Zhao X, Zhu H, Zhou H. (2013). Mitochondrial DNA Analysis of M21 Xiongnu Tomb in Hulaha Valley of Mongolia. [Meng gu guo hu la ha shan gu M21 hao xiong nu mu zhu de xian li ti DNA fen xi]. *Research of China’s Frontier Archaeology*(1), 309-314.

Cai DW, Zhu SQ, Zhao X, Sun WL, Zhang QC, Chen YZ, Zhu H, Zhou H. (2016). Molecular Archaeological Research on Human Remains from the Khulhiin Am and Khundiin Khooloi Sites in Mongolia During the Mongol-Yuan Period. [Meng gu guo Khulhiin Am he Khundiin Khooloi meng yuan shi qi gu ren fen zi kao gu yan jiu]. *Research of China’s Frontier Archaeology*(1), 291-296.

Chen WD, Lei Y, Zheng WQ, Zheng LH, Rang HL, Zhang DZ, Chen DA, Zhao J. (2021). Brief Investigation Report of the Bronze Age Sites in Central and Northern Vietnam. [Yue nan zhong bei bu qing tong shi dai yi zhi diao cha jian bao]. *Sichuan Cultural Relics*(1), 4-18.

Chen YZ, Song GD, Yue GM, Sarrenbillige, Jian Z, А.Ochir, B.Ankhbayar, Ma J. (2016). The Excavation of Uighur Graveyard No.6 at Khirgisiin Khooloin,Arkhangai Province,Mongolia. [Meng gu guo hou hang ai sheng he lie ke si hao lai shan gu 6 hao hui he mu yuan fa jue jian bao]. *Cultural Relics*(4), 32-40.

Ding Y. (2014). Preliminary Study on the Old City Site in Malindi,Coastal Province,Kenya. [Ken ni ya bin hai sheng ma lin di lao cheng yi zhi de chu bu yan jiu]. *Cultural Relics in Southern China*(4), 130-138.

Ding Y. (2017). Chinese Ceramics and East African Pillar Tombs. [Zhong guo ci qi yu dong fei zhu mu]. *Palace Museum Journal*(5), 133-145.

Ding Y, Lu ZY, Miao YF, Zhao HQ, Gulmira M, Turaly T, Zhazira K. (2017). Survey and Excavation of the Rahat Site. [La ha te gu cheng yi zhi diao cha yu shi jue]. *Archaeology and Cultural Relics*(6), 121-122.

Ding Y, Qin D. (2016). Chinese Porcelains Unearthed from the Ungwana Site in Kenya. [Ken ni ya wu gua na yi zhi chu tu de zhong guo ci qi]. *Archaeology and Cultural Relics*(6), 26-46.

Feng EX, Aerjin. (2006). The Gains of Troysky Graveyard's Excavation of Russia in 2004. [E luo si te luo yi ci ji mu di 2004 nian fa jue de shou huo]. *Research Of China's Frontier Archaeology*(00), 211-215.

Li J, Zhang Y, Zhao Y, Chen Y, Ochir A, Sarenbilige, Zhu H, Zhou H. (2018). The Genome of an Ancient Rouran Individual Reveals an Important Paternal Lineage in the Donghu Population. *American Journal of Physical Anthropology, 166*(4), 895-905. doi:10.1002/ajpa.23491

Li MR, Li XW, Ramos J. (2020). Brief Report on the Excavation of the Large Tomb No.3 in the North Complex of the 8n-11 Elite Compound at the Copan Site,Honduras. [Hong du la si ke pan yi zhi 8N-11 hao gui zu ju zhi bei ce jian zhu 3 hao da mu]. *Archaeology*(8), 38-51+32.

Li XW. (2018). The Sculptures of Mexican Year Signs and Crossed Torches of the Aristocrat Residential Site 8n-11 at Copan Site in Honduras. [Hong du la si ke pan yi zhi 8N-11 hao gui zu ju zhi "mo xi ge ji nian" he jiao cha huo ju diao ke]. *Archaeology*(10), 101-109.

Li XW, Ramos J, Peng XJ, Fu YX, Guo ZW, Zhou ZY, Zhang D, Jia XB. (2017). The Architectural Remains of the Later Period to the North of the Elite Compound Site 8n-11 at Copan Site in Honduras. [Hong du la si ke pan yi zhi 8N-11 hao gui zu bei ce wan qi jian zu]. *Archaeology*(9), 39-44+126+145-158+122.

Liang HL, Xie F. (2016). Report on the Ancient Kraskino City in Russia's Primorsky Region,2011. [2011 nian e luo si bin hai bian jiang qu ke la si ji ruo cheng zhi kao gu kan tan bao gao ]. *Northern Cultural Relics*(2), 29-34.

Liang Y, Li WW, Han L. (2021). Discussion of the Yuezhi Culture in North Bactria. [Lun bei ba ke te li ya de yue zhi wen hua]. *Archaeology*(9), 5-108.

Liang Y, Li WW, Pei JL, Tang YP. (2018). Brief Report on the 2017 Excavation of the Rabat Cemetery in Boysun City, Uzbekistan. [Wu zi bie ke si tan bai song shi ba la te mu di 2017 nian fa jue jian bao]. *Cultural Relics*(7), 4-30+32+31.

Liang Y, Liu B, Nuriddin S, Wang R, Zhao DY, Wu C, Su h, Xiao GQ, Xia R, Xing DL, Zhang RY, Abdullo S, Asliddin K, Islamjon A, Mehrobiddin S, Han L. (2020). Preliminary Report on the Survey and Excavation of the Kaskar Cemetery in Tadzhikistan in 2018. [Ta ji ke si tan ka shi ka er mu di 2018 nian diao cha fa jue jian bao]. *Archaeology and Cultural Relics*(3), 13-26.

Liu KY, Zhao DY, Liang Y, HASANOV M, Wang JX, MAKSUDOV F, Lin X. (2020). Study on the Life Style of the Ancestors Buried in Rabat Cemetery Based on Stable Isotope Analysis. [Ji yu wen ding tong wei su fen xi de la ba te mu di xian min sheng huo fang shi]. *Scientia Sinica(Terrae), 50*(11), 1611-1617.

Liu Y, Qin DS, Kiriama H. (2012). The Chinese Porcelains Unearthed at Gedi Ruins in Coast Province, Kenya. [Ken ni ya bin hai sheng ge di gu cheng yi zhi chu tu zhong guo ci qi]. *Cultural Relics*(11), 37-60+31.

Qiao L, Li YQ. (2003). Archaeological Excavation of the Chau Say in Ankor. [Wu ge yi ji zhou sha shen miao kao gu bao gao]. *Acta Archaeologica Sinica*(03), 427-458+467-474.

Qiao L, Wang YL, Yu JL. (2018). The Assorting of the New-Discovered Artifacts at Chau Say Tevoda in Angkor, Cambodia. [Jian pu zhai wu ge gu ji zhou sha shen miao xin fa xian yi wu zheng li jian bao]. *Archaeology*(4), 58-68.

Qin DS, Xu HF, Majorah M. (2014). The Chinese Porcelains Salvaged from Mombasa Wreck in Kenya. [Ken ni ya meng ba sa ta na hao shen chen chu shui de zhong guo ci qi]. *Palace Museum Journal*(2), 6-24.

Ren M. (2014). Archaeological Surveys in Tajikistan and Uzbekistan: Copper and Stone Age to Hellenistic Age. [Ta ji ke si tan, Wu zi bie ke si tan kao gu diao cha ---- tong shi bing yong shi dai zhi xi la hua shi dai]. *Cultural Relics*(7), 54-67.

Ren M. (2015). Archaeological Investigations in Tajikistan and Uzbekistan——Pre-Kushan Period to Post-Kushan Period [Ta ji ke si tan, Wu zi bie ke si tan kao gu diao cha --- qian gui shuang shi dai zhi hou gui shuang shi dai]. *Cultural Relics*(6), 17-33.

Rong TY, Telbayr, Bator O, Tsering D, Batu N, Bals, Bayar E. (2021). Brief Report on the 2018-2019 Excavation of the Airagiin Gozgor Cemetery in Jargalant Sum,Mongolia. [Meng gu guo ji er ga lang tu su mu ai er gen ao bao mu di 2018-2019 nian fa jue jian bao]. *Archaeology*(11), 48-63.

Sarrenbillige, Cheng PF, Song GD, Cao JE, Chen YZ, A.Ochir, B.Ankhbyer, G.Mandakhbayar. (2020). Brief Report on the Excavation of Khermental City Site in Ugiinuur Sum, Arkhangai Province, Mongolia. [Meng gu guo hou hang ai sheng wu gui nuo er su mu he ri men ta la cheng zhi fa jue jian bao]. *Archaeology*(5), 20-37+480.

Siu I, Henderson J, Qin D, Ding Y, Cui J. (2021). A Study of 11th-15th Centuries Ad Glass Beads from Mambrui, Kenya: An Archaeological and Chemical Approach. *Journal of Archaeological Science: Reports, 36*, 102750.

Siu I, Henderson J, Qin D, Ding Y, Cui J, Ma H. (2020). New Light on Plant Ash Glass Found in Africa: Evidence for Indian Ocean Silk Road Trade Using Major, Minor, Trace Element and Lead Isotope Analysis of Glass from the 15th—16th Century Ad from Malindi and Mambrui, Kenya. *PLoS One, 15*(8), e0237612. doi:10.1371/journal.pone.0237612

Song GD, Chen YZ, Bao WS. (2016). An Analysis of the Turkic Luni Text Tile Excavated from the Uighur Graveyard No.6 at Khirgisiin Khooloin,Arkhangai Province,Mongolia. [Meng gu guo he lie ke si hao lai shan gu 6 hao hui he mu yuan chu tu tu jue lu ni wen wa kao xi]. *Cultural Relics*(4), 51-53.

Sun ZY, Ding Y, Lu ZY, Miao YF, Zhao HQ, Liu JF, Qi YT, Wu XC, Gulmira M, T.H.Toybaev, Turaly T, Zhazira K. (2020). Preliminary Report on the Excavation of Tomb No.M1 at an Early Iron-Age Cemetery to the Southeast of the Rahat Site in Isek,Kazakhstan. [Ha sa ke si tan yi sai ke la ha te dong nan mu di zao qi tie qi shi dai M1 fa jue jian bao]. *Archaeology and Cultural Relics*(3), 3-12+12.

Tang YP, Li WW, Liang Y, Wang JX. (2020). Brief Report on the 2018 Excavation of the Rabat Cemetery in Boysun City, Uzbekistan. [Wu zi bie ke si tan bai song shi la ba te mu di 2018 nian fa jue jian bao]. *Archaeology*(12), 53-80+52.

Tang ZW, Feng EX, Zhang SQ. (2008). Preliminary Study on the Palaeoenvironmet in the Area of Troitsky Burial Ground of Ivanovka District in Amurskaya,Russia. [E luo si a mu er zhou yi wan nuo fu ka qu te luo yi ci ji mu di gu huan jing chu tan]. *Quaternary Sciences, 28*(6), 1166-1174.

Tarra, Chen YZ, Song GD, Yue GM, Sarrenbillige. (2009). Excavation of a Xiongnu Tomb on the Khulhiin Am Site in Khotont Sum,Arkhan- Gau Province,Mongolia. [蒙古国后杭爱省浩腾特苏木胡拉哈山谷匈奴墓的发掘]. *Archaeology*(6), 50-56+110-111.

Wang JX, Masksudov F, Liang Y, Liu B, Muttalib H, Xing DL, Han L, Komil A, Nasibillo KSK, Guo XX. (2020a). Preliminary Report on the Excavation of Tomb No.M11-2 at the Sazagan Site in Samarqand,Uzbekistan. [Wu zi bie ke si tan ma er han shi sa zha gan yi zhi M11-2 fa jue jian bao]. *Archaeology and Cultural Relics*(3), 27-36.

Wang SH. (2016). Research and Analysis on the Architectural Structure of Ta Keo in Angkor, Cambodia. [Jian pu zai wu ge cha jiao si jian jian zhu gou zao de yan jiu yu jie xi]. *Northern Cultural Relics*(4), 46-52.

Wang XM, Kolchenko; V, Zhang JL, Tian YQ, Hu CB, Yan ZY, Kiy E, Shao AD, Huang XJ, Yan JY, Liu J, Liu W, Bai WS. (2020b). Preliminary Report on the Excavation of the Western Section of the Buddhist Temple at the Rechka Site in Kyrgyzstan. [Ji er ji si si tan hong he gu cheng xi ce fo si yi zhi 2018-2019 nian du fa jue jian bao]. *Archaeology and Cultural Relics*(3), 37-51+129.

Xi TY. (2019). Archaeological Surveys in Tajikistan and Uzbekistan:On the Sogdian Period. [Ta ji ke si tan, Wu zi bie ke si tan kao gu diao cha ---- Su te shi qi]. *Cultural Relics*(1), 44-66+41.

Yu JL, Wang YL, Qiao L. (2017). The Archaeological Survey to the Remains Nearby the Ta Keo Site in Angkor, Cambodia. [Jian pu zhai wu ge cha jiao si zhou bian yi zhi kao gu diao cha jian bao]. *Archaeology*(9), 59-72+52.

Yusuf R, Xi TY, Liang Y. (2018). Preliminary Report on the Excavation of Tomb No.M11 at the Sazagan Site in Samarqand,Uzbekistan. [Wu zi bie ke si tan sa ma er han shi sa zha gan yi zhi M11 fa jue jian bao]. *Cultural Relics*(7), 31-41.

Zhang LR, Тишкин АА, Грушин СП, Серегин НН. (2017). The Excavation of Settlement Kalyvanskoe-Ⅰ in Zmeinogorsk, Russia. [E luo si she shan shi ka le wang hu 1 hao yi zhi de fa jue]. *Archaeology*(9), 14-21+12.

Zhang QC, Feng EX, Zhu H. (2008). A Research on the Ancient Human Skulls from the Troitskiy Cemetery in Far-Eastern Region of Russia. [E luo si yuan dong di qu te luo yi chi ji mo he mu di ren gu yan jiu]. *Acta Anthropologica Sinica*(2), 120-128.

Zhang QC, Feng EX, Zhu H. (2009). Paleodiet Studies Using Stable Carbon and Nitrogen Isotopes from Human Bone:An Example from the Troitskiy Cemetery of Mohe,Far-Eastern Area of Russia. [E luo si yuan dong di qu te luo yi chi ji mo he mu di ren gu de wen ding tong weisu fen xi]. *Acta Anthropologica Sinica*(3), 300-305.

Zhang W, Zhao JB, Deng QJ, Meng YZ, Wang J, WAng YC, Er J, Qiu YS, Lin GC, Wang GY, Zhu B, Lou JL, Yang ZL, Xie GW, Zhai Y, Feng L, Zhang H, Zeng J, Zhan B, Kai S. (2014). 2013 Excavation Report of Ngomeni Ras Shipwreck Site in Malindi, Kenya. [Ken ni ya ma lin di ao mei ni jiao chen chuan yi zhi 2013 nian du shui xia kao gu fa jue jian bao]. *Journal of National Museum of China*(9), 6-23.

Zhao CQ, Chen CS, Liu GX, Xiao HY, Yan ZB. (2005). A Summary on the Archaeological Exploration,2002,in the Maritime Region of Russia. [E luo si bin hai di qu 2002 nian kao gu kao cha ji yao]. *Archaeology*(8), 74-90+104+102.

Zhao JB, Zhu B, Meng YZ, Zhai Y. (2012). Major Discoveries of Sino-Kenyan Cooperative Underwater Archaeological Survey in Coastal Areas of Kenya in 2010. [2010年度中肯合作肯尼亚沿海水下考古调查主要收获]. *Journal of National Museum of China*(8), 88-100.

Zhou LG, Lan WL, Liu B, Nie F, Ren X, Deng XB, Zhou RS, Sun K, Zhou T, Bao WK, E.Mijiddorj, B.Galbadrakh, J.Erdene. (2021). 2017-2019 Excavation of Gol Mod 2 in Arhangay Aymag,Mongolia. [Meng gu guo hou hang ai sheng gao le mao du 2 hao mu di 2017-2019 nian kao gu fa jue jian bao]. *Huaxia Archaeology* (6), 39-53.

Zhou LG, Zhou RS, Nie F, Lan WL, Wang J, Ren X, D.Erdenebaatar. (2018). The Excavation of the Satellite Tome M189 in Gol Mod Cemetery No.2 in Arkhangai Province,Mongolia. [Meng gu guo hou hang ai sheng gao le mao du 2 hao mu di M189 pei zang mu fa jue jian bao]. *Huaxia Archaeology*(2), 34-44+32+129.

Zhu YS, Liu T, Ai LJ, He SL. (2017). Archaeological Survey and Excavation of the Mingtepa Site in Andijan Region, Uzbekistan. [Wu zi bie ke si tan an ji yan zhou ming tie pei cheng zhi kao gu kan tan yu fa jue]. *Archaeology*(09), 22-38+22.

Zhu YS, Matbabaev BH. (2019). Archaeological Survey and Excavation of the Mingtepa Site in Andijan Region, Uzbekistan. *Chinese Archaeology, 19*(1), 150-162.
